# Biofluid Proteomic Biomarker Discovery in a Large Animal Model of CLN2 Neuronal Ceroid Lipofuscinosis

**DOI:** 10.3390/biology15191761

**Published:** 2026-10-02

**Authors:** Brandie R. Morgan-Jack, Grace R. Kick, Zhen Lyu, Trupti Joshi, Brian Mooney, Joan R. Coates, Juri Ota-Kuroki, Martin L. Katz, Rebecca E. H. Whiting

**Affiliations:** 1 Department of Ophthalmology, School of Medicine, University of Missouri, Columbia, MO 65212, USA; morganbr@health.missouri.edu (B.R.M.-J.); katzm@health.missouri.edu (M.L.K.); 2 Department of Pediatrics, Washington University in St. Louis, St. Louis, MO 63110, USA; kick@wustl.edu; 3 Department of Biomedical Sciences, Joan C. Edwards School of Medicine, Marshall University, Huntington, WV 25701, USA; lyuz@marshall.edu (Z.L.); joshitr@marshall.edu (T.J.); 4 Christopher S. Bond Life Science Center, University of Missouri, Columbia, MO 65211, USA; 5 MU Institute for Data Science and Informatics, University of Missouri, Columbia, MO 65211, USA; 6 Department of Biomedical Informatics, Biostatistics and Medical Epidemiology, School of Medicine, University of Missouri, Columbia, MO 65211, USA; 7 Gehrke Proteomics Center, University of Missouri, Columbia, MO 65211, USA; mooneyb@missouri.edu; 8 Department of Veterinary Medicine & Surgery, College of Veterinary Medicine, University of Missouri, Columbia, MO 65211, USA; coatesj@missouri.edu (J.R.C.); otaj@missouri.edu (J.O.-K.)

**Keywords:** canine model, neuronal ceroid lipofuscinosis, CLN2 disease, proteomics, protein biomarkers, cerebrospinal fluid, plasma, tear fluid, neurodegeneration

## Abstract

CLN2 disease is a fatal lysosomal storage disorder caused by deficiency of the lysosomal enzyme tripeptidyl peptidase 1 (TPP1), resulting in progressive neurodegeneration and non-neuronal pathology. While enzyme replacement therapy has improved disease management, there remains a critical need for objective molecular biomarkers capable of monitoring disease progression and therapeutic response. The purpose of this study was to utilize a canine model of CLN2 disease to investigate proteomic changes in cerebrospinal fluid (CSF) and in minimally invasive biofluids tears and plasma to identify candidate disease biomarkers. Untargeted proteomic analysis was conducted using these biofluids from unaffected controls and CLN2-affected dogs at presymptomatic, symptomatic, and advanced disease stages. CSF demonstrated the greatest degree of proteomic alterations over the disease progression. Proteins associated with cytoskeletal organization, synaptic signaling, lysosomal biology, mitochondrial metabolism, lipid homeostasis, and inflammatory responses were significantly altered. Tear fluid demonstrated overlapping biological themes with CSF, particularly involving mitochondrial metabolism, lysosomal function, and lipid-associated pathways. In contrast, plasma proteomic alterations were comparatively modest but supported systemic changes associated with extracellular signaling, inflammation, and lipid metabolism. Collectively, these findings demonstrate progressive and coordinated proteomic alterations across multiple biofluids in CLN2 disease that can serve as candidate biomarkers of disease progression.

## Simple Summary

CLN2 disease is a rare inherited childhood disorder that causes progressive damage within and outside of the nervous system. Although treatment can slow disease progression, sensitive biological markers are needed to monitor disease and determine how well therapies are working. In this study, we examined changes in proteins present in three biofluids: cerebrospinal fluid (fluid that surrounds the brain and spinal cord), blood plasma, and tears from dogs with CLN2 disease. Changes were detected before the appearance of clinical signs and became more extensive as the disease advanced. Altered proteins were involved in such processes as nerve cell structure, cellular waste removal, energy production, lipid metabolism, and immune responses. Cerebrospinal fluid had the most prominent changes and had several commonalities with tear protein alterations. Plasma showed the most modest alterations but still displayed changes consistent with disease mechanisms. These findings demonstrate that CLN2 disease produces measurable molecular changes across multiple body fluids that can be used to identify proteins that may be useful for monitoring disease progression.

## 1. Introduction

Inborn errors of metabolism (IEMs) are a large class of hereditary diseases generally resulting from deficiencies in enzymes, causing blockages in associated metabolic pathways, toxic accumulations of metabolic intermediates, and deficiencies of downstream metabolites [1]. Among the IEMs is a group of lysosomal storage diseases known as the neuronal ceroid lipofuscinoses (NCLs). Forms of NCL have been associated with variants in at least 13 genes [2,3,4]. The CLN2 form of NCL is caused by mutations in the *TPP1* gene that encodes the soluble lysosomal enzyme tripeptidyl peptidase 1 [5]. CLN2 disease usually has an onset in early childhood and is typically fatal by the early teenage years. Symptoms include cognitive and motor deficits, seizures, and visual deterioration leading to blindness [6]. Pathologically, the disease is characterized by progressive brain atrophy and retinal degeneration, and by abnormal intracellular accumulation of autofluorescent lipopigments in both neuronal and non-neuronal tissues [7].

Dogs with a spontaneous null mutation in the *TPP1* gene experience a highly predictable disorder that closely recapitulates human CLN2 disease over 10 to 11 months [8] and have proven to be a valuable translational model. The model was used in preclinical studies that were essential in the development of cerliponase alfa [9,10,11], the only FDA-approved treatment currently available for CLN2 disease [12], and in supporting the current phase I/II intravitreal enzyme replacement therapy (ERT) clinical trial [13,14] (ClinicalTrials.gov ID: NCT05152914). As CLN2-affected dogs only survive to 10 to 11 months of age, studies spanning the entire course of the disease arefeasible.

In order to monitor the therapeutic efficacy of existing and potential new treatments for CLN2 disease, there is a need for objective biomarkers of disease status. Currently, the only tools available for disease monitoring in CLN2 patients include clinical rating scales that assess feeding and motor abilities, gait, language development, vision, and seizures [15,16]. While informative, these parameters may not be sensitive enough to fully inform short-term treatment decisions. Measures that reflect early disease progression at a molecular level, such as protein alterations in easily accessible biofluids, would be useful in timely assessment of the efficacy of therapeutic interventions [1,17].

Proteomic analyses of brain tissue and cerebrospinal fluid (CSF) collected on autopsy from human patients with CLN2 disease identified proteins that were consistently altered, in addition to several other proteins that warranted further investigation [18,19]. While these studies identified candidate biomarkers, a noted limitation was that age-matched controls were not available, and data from analysis of human subject samples could be confounded by postmortem artifact and only reflected terminal disease-related changes. Analysis of blood plasma from patients with multiple forms of NCL identified 7 potential biomarkers that differed significantly between NCL patients and controls [16].

It would be highly beneficial to monitor biomarkers that reflect central nervous system pathology without the need for CSF collection. Blood plasma and tear fluid are two alternative minimally invasive candidates, and disease-related proteome alterations in these fluids have been reported in several neurodegenerative diseases including multiple sclerosis (MS) and Parkinson’s disease [20,21,22,23]. For example, changes in the protein makeup of tears from patients with MS were found to correlate with similar alterations in the CSF, demonstrating a direct crosstalk between the two fluids [20]. Additionally, recent studies have highlighted tear fluid as a biologically active and clinically accessible biofluid that reflects local and systemic molecular processes, with proteins involved in inflammation, oxidative stress, immune regulation, and cellular homeostasis, all of which are implicated in neurodegeneration [24].

The goal of this exploratory, early investigation study was to complement previous NCL proteomic investigations by identifying potential protein biomarkers of disease progression in CSF from the canine model of CLN2 disease and to identify candidate protein biomarkers in less-invasively collected biofluids (tear fluid and plasma). To achieve this, we performed untargeted proteomic analysis of the three biofluids from healthy controls and CLN2-affected dogs at presymptomatic, symptomatic, and late-stage disease time points.

## 2. Materials and Methods

### 2.1. Animals

Dogs in this study were untreated, purpose-bred long-haired miniature Dachshunds. Affected dogs (n = 8, 2 female and 6 male) were homozygous for a c.325delC null frameshift variant in *TPP1* [8]. Normal, unaffected dogs were heterozygous carriers for the wild-type and c.325delC alleles (n = 9; 6 female and 3 male). Carrier females were bred to carrier or affected males with no common ancestor between each breeding pair for at least the two preceding generations. Dogs were assigned to study groups on a rolling basis, and small sample sizes were employed based on previous studies demonstrating robust differences between CLN2-affected dogs and healthy controls. Dogs were implanted with microchips for identification and genotyped at the *TPP1* variant locus using an allelic discrimination assay that distinguishes the wild-type and the disease-associated c.325delC *TPP1* alleles [8]. Dogs were housed at the University of Missouri, received standard husbandry and veterinary care, and were socialized daily. All procedures were approved by the University of Missouri Animal Care and Use Committee.

### 2.2. Neurological Examination

A neurological examination was performed by a board-certified veterinary neurologist or veterinary neurology specialty intern in training prior to enrollment in the study. The neurological examination was repeated for each dog regularly throughout the study period and consisted of observation of mentation, posture and gait, cranial nerve evaluation, postural reaction testing, spinal reflexes, spinal palpation and pain assessment. Dogs with neurologic abnormalities unrelated to CLN2 disease were excluded from the study.

### 2.3. Ophthalmologic Examination

Each dog received a complete ophthalmic examination by a board-certified veterinary ophthalmologist prior to enrollment in the study and monthly thereafter. The exam included Schirmer tear test-1 (Merck Animal Health, Summit, NJ, USA), Tonovet tonometry (TonoVet^®^; Icare Finland Oy, Vantaa, Finland), corneal application of fluorescein stain (Bio-Glo; HUB Pharmaceuticals, LLC, Scottsdale, AZ, USA), slit-lamp biomicroscopy (Kowa SL-17; Kowa Co., Ltd., Tokyo, Japan), and indirect ophthalmoscopy with a binocular headset and a 28D condensing lens (Keeler Vantage; Keeler Instruments, Inc., Mavern, PA, USA and Volk Optical Inc., Mentor, OH, USA, respectively). The pupils were dilated with topical tropicamide 1% ophthalmic solution USP (Akorn, Lake Forest, IL, USA) for fundic evaluation. Dogs with ophthalmic abnormalities unrelated to CLN2 disease were excluded from the study.

### 2.4. Biofluid Collections

CSF and plasma samples from control and affected dogs were collected and analyzed at 3 time points: pre-symptomatic (3.6–4.2 months of age), symptomatic (6.6–8.6 months of age), and late-stage disease (9.3–11.2 months of age). Age-matched tear fluid samples from unaffected dogs were analyzed at ages corresponding to the pre-symptomatic and symptomatic time points. For tear collection at the late-stage time point due dogs had to be sedated due to progression of disease-relatedmyoclonus. Due to the nature of the study, masking of study personnel was not feasible. To minimize potential bias, standardized procedures were implemented for sample collection and processing. Sample numbers for each biofluid and time point are shown in Table 1.

Whole blood was collected from the jugular or a peripheral vein into vacutainer tubes containing K2EDTA as an anticoagulant. Blood samples were centrifuged for 10 min at 1591× *g*, and plasma was separated and stored at −80 °C.

Tear fluid was collected using ophthalmic sponges placed under the lower lid for 60 s [25]. Immediately afterward, the sponges were frozen at −80 °C. Tear samples collected over 4 consecutive days for each time point were pooled to ensure sufficient quantities of protein for analysis. Prior to analysis, sponges were thawed briefly at room temperature, and tear fluid was recovered using a “piggyback” method by centrifuging at 3900× *g* for 2 min [25,26]. Samples from both eyes were pooled, lyophilized, and resuspended in 50 µL of ultrapure water.

CSF collection was performed by a board-certified veterinary neurologist (JRC) or a veterinary neurology specialty intern in training, while the dog was under general anesthesia. A 22-gauge 1.5-inch spinal needle was used to percutaneously puncture the dorsal atlanto-occipital membrane into the cerebellomedullary cistern. Approximately 0.2 to 0.5 mL of CSF was collected using gravity flow and stored at −80 °C. Prior to analysis, the total volume of collected CSF was lyophilized and resuspended in 500 µL of ultrapure water.

### 2.5. Sample Preparation and Mass Spectrometry

Untargeted proteomic analyses were performed at the University of Missouri Gehrke Proteomics Center. Protein in reconstituted tear fluid was precipitated by addition of 3× the sample volume of acetone with 5% *w*/*v* trichloroacetic acid and stored overnight at −20 °C. Samples were centrifuged for 10 min at 16,000× *g*, and the resulting pellet was dissolved in 25 µL of 6 M urea/2 M thiourea/100 mM ammonium bicarbonate, pH 8.0 (AmBic).

Plasma and CSF samples were depleted of highly abundant proteins with perchloric acid [27]. Neat plasma was diluted 1:10 in ultrapure water. 15% perchloric acid was added to samples (final concentration of 0.3%), and samples were mixed vigorously and chilled at −20 °C for 15 min, followed by centrifugation at 16,000× *g* for 15 min at 4 °C. The pellet was discarded, and 1% trifluoroacetic acid (TFA) was added to the supernatant depleted fraction (final concentration of 0.1%). Samples were loaded onto a preconditioned (sequential MeOH & 0.1% TFA) SPE Oasis HLB 96-well plate, washed with 0.1% TFA, and eluted with 90% acetonitrile/0.1% TFA. Eluted samples were dried overnight by speed vacuum concentration. Samples were then resuspended in 25 µL of 6 M urea/2 M thiourea/100 mM ammonium bicarbonate, pH 8.0 (AmBic).

All samples were reduced with dithiothreitol (DTT) (final concentration in 100 mM AmBic was 5 mM for plasma and CSF and 10 mM for tears) for 1 h at room temperature with agitation. Samples were then alkylated with iodoacetamide (IAA) (final concentration in 100 mM AmBic was 14 mM for plasma and CSF and 40 mM for tears) for 1 h at room temperature with agitation. Tear samples were again incubated with DTT (equivalent volume as IAA) for 20 min at room temperature with agitation. Protein concentrations were determined by Pierce BCA assay; total protein was normalized in 6 M urea/2 M thiourea/100 mM AmBic, and urea concentration was reduced to ~0.6 M with ultrapure water. Samples were digested with 0.5 µg trypsin (target ratio of 1:50, trypsin: protein) overnight at 37 °C. The following day, an additional aliquot of trypsin was added, and samples were incubated for an additional 4–6 h at 37 °C. Tear fluid peptides were concentrated and desalted with Pierce™ C18 tips, lyophilized, and resuspended at 1 µg/µL. For plasma and CSF, 1 µg of protein was loaded onto C18-Evotips.

LCMS DIA-PASEF data were collected on a Bruker timsTOF-Pro2 via a Bruker CaptiveSpray source. 1 µg of protein was loaded onto a Pepsep25 (tear fluid) or Pepsep15 (plasma and CSF) column. LC (Bruker nanoElute 2) gradient conditions for tear fluid analyses were as follows: A: 0.1% formic acid in water, B: 99.9% acetonitrile, 0.1% formic acid; 3%B, 20 min gradient to 17%B, 17–25%B over 10 min, 25–37%B over 10 min, 37–80%B over 6 min, 3 flushing cycles (80%B–40%B for 2 min), 3%B for 2 min, hold for 4 min to equilibrate. LC gradient conditions for CSF and plasma used the 30SPD Evosep method (30 samples per day, 44 min, Evosep One HPLC). MS data were collected in positive-ion data-independent acquisition PASEF mode [28] over an *m*/*z* range of 300.5 to 1349.5 and a mobility range of 0.65 to 1.45. Fifty DIA windows were collected (of varying *m*/*z* width) for a total cycle time of 2.76 s. An ion-mobility-based collision energy was used with 20 eV at 0.85 and 59 eV at 1.30. Data were copied to Spectronaut V19 and searched against the NCBI-Dog FASTA database (150,751 entries; last update 03/2019) using the directDIA approach with default parameters (trypsin as enzyme, 2 missed cleavages allowed; 20 ppm mass error on precursor, 0.1 Da mass error on CID MSMS fragments; carbamidomethyl-Cys fixed modification; oxidized-Met and protein N-terminus acetylation as variable modifications). Protein abundance was reported as the sum of peptide fragment intensities for each protein (top 3 peptides per protein).

### 2.6. Differential Protein Abundance Analyses

Due to the exploratory nature of this study, the primary differential abundance results were based on raw *p*-values rather than FDR-adjusted significance. FDR-adjusted results can be found in the respective results tables and Supplementary Tables. Differential protein abundance analysis was performed using the edgeR package in R (version 4.0.16) [29]. Raw protein abundance values (PG.Quantity) were extracted for each sample, forming a matrix with proteins as rows and samples as columns. As a post-hoc quality-control step, principal component analysis (PCA) was performed on the CSF, plasma, and tears proteomics datasets (log2-transformed protein intensities, z-score; proteins quantified in ≥80% of samples retained) to screen for multivariate outliers, flagged as samples >3 SD from the overall sample centroid on PC1/PC2. For proteins retained after filtering, missing intensity values were imputed using the k-nearest neighbors algorithm (k = 10) via the impute R package (version 1.86.0). For each data type and disease stages, the filtered and imputed intensity matrix was used to create a DGEList object with the corresponding group information, with internal normalization explicitly disabled (norm.method = none) to avoid inappropriate count-based adjustments. Sample-specific scaling factors were calculated as the column-wise sum of intensities, and their log-transformed values were supplied as offsets to the model to account for differences in overall protein abundance between samples. Dispersion estimates were calculated using the robust quasi-likelihood (QL) framework (estimateDisp with robust = TRUE) to better accommodate the continuous nature of the data. Differences in protein abundance between CLN2-affected and control groups were assessed using a quasi-likelihood F-test (glmQLFTest). Proteins were considered differentially abundant if they had a *p*-value < 0.05 and a log2 fold change >0.585 or <−0.585 (corresponding to a 1.5-fold change).

### 2.7. Functional Enrichment Analyses

Functional enrichment analysis was performed for each set of differentially abundant proteins (DAPs) for each comparison. Gene Ontology (GO) enrichment analyses for Biological Process (BP), Molecular Function (MF), and Cellular Component (CC) were conducted using g:Profiler (version e113_eg59_p19_2063ef92; database updated on 23 May 2025) [30]. Multiple testing correction was applied using the Benjamini–Hochberg method, and enriched pathways and GO terms with an adjusted *p*-value < 0.05 were considered statistically significant.

### 2.8. Multi-Omics Integrative Correlation Analyses

The mixOmics R package (version 6.22.0) was used to integrate differentially abundant proteins across CSF, plasma, and tear datasets for comparisons between affected and control groups [31]. Protein data consisted of DAPs identified at the pre-symptomatic and symptomatic time points for each data type, defined by a *p*-value < 0.05 and an absolute log2 fold change ≥0.585. Proteins meeting these criteria in at least one comparison were retained, and duplicate entries were removed to generate the final feature set for integrative analysis.

Data integration and classification were performed using Data Integration Analysis for Biomarker discovery using Latent variable approaches for Omics studies (DIABLO). N-integration sparse partial least squares discriminant analysis (sPLS-DA) was applied to identify multi-omics signatures composed of highly correlated proteins across CSF, plasma, and tear datasets, enabling the identification of coordinated abundance patterns associated with disease status across biofluids.

Model tuning was performed prior to interpretation of the DIABLO results. Feature selection was applied using tune.block splsda() with 5-fold cross-validation (10 repeats), minimizing the balanced error rate. Since 9 of 11 animals contributed samples at both pre-symptomatic and symptomatic stages, classification performance was evaluated using Leave-One-Animal-Out cross-validation.

Protein correlations across CSF, plasma, and tear datasets were assessed, and only protein pairs with an absolute correlation coefficient ≥0.7 were retained. Correlation matrices were visualized using circos plots generated with the circlize R package (version 0.4.15).

### 2.9. Statistical Analyses

Statistical analyses were performed in R [31]. Differences in protein abundance between affected and control groups were assessed at each time point using a negative binomial modeling framework [32]. Proteins with a raw *p*-value < 0.05 and a log2 fold change >0.585 or <−0.585 between CLN2-affected and unaffected dogs were considered differentially abundant. Functional enrichment analyses were conducted using g:Profiler with Benjamini–Hochberg adjustment (adjusted *p*-value < 0.05) [30,33]. Integrative analyses across CSF, plasma, and tear datasets were performed using latent variable–based approaches, with a correlation cutoff of 0.7 [34,35].

## 3. Results

### 3.1. CLN2-Related Disease Progression

Neurologic deficits observed in the CLN2-affected dogs enrolled in this study followed a similar progression to that reported previously [9,36]. At 4 months of age (pre-symptomatic), the neurologic examination was normal. By 8 months (symptomatic stage), significant neurologic deficits included cerebellar ataxia, intention and resting tremors, postural reaction deficits, myoclonic jerks, and menace response deficits. By 10 months of age (late-stage), the neurologic deficits had markedly progressed, and seizure activity was controlled with antiseizure drug therapy; CLN2-affected dogs reached end-stage disease (inability to eat without significant assistance, loss of visual tracking, severe mentation defects, and medication-refractory myoclonic jerks) and were euthanized for humane reasons between 10 and 11 months of age.

CLN2-affected dogs in this study showed ophthalmic, visual behavior, and retinal changes consistent with previous reports [37,38,39], including multifocal retinal detachments [40]. Ophthalmic changes included progressive deficits in retinal structure and function beginning early in the disease course around 5 months of age, followed by changes in visual behavior later in the disease (symptomatic and late-stage).

### 3.2. Cerebrospinal Fluid (CSF) Protein Alterations

**Figure 1:**
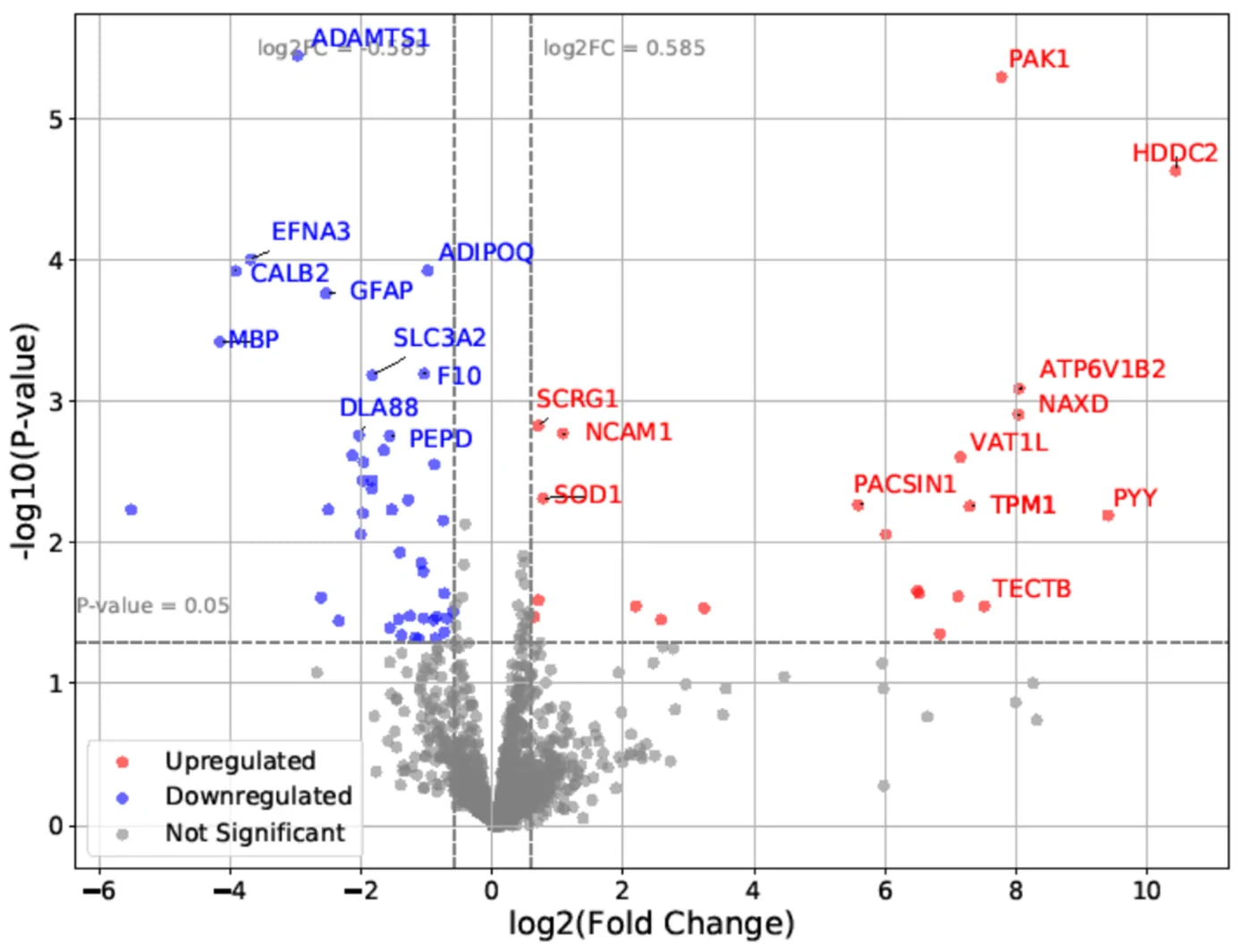
Differentially abundant proteins in CSF from CLN2-affected dogs at the presymptomatic time point. Volcano plot showing differential protein abundance between CLN2-affected and age-matched control dogs. The x-axis represents log2 fold change (log2FC), and the y-axis represents −log10(*p*-value). Proteins meeting significance thresholds (raw *p* < 0.05 and log2 fold change >0.585 or <−0.585) are highlighted in red (increased abundance) or blue (decreased abundance), while non-significant proteins are shown in gray. Each dot indicates a specific protein that was identified. Identities of the 10 most significantly altered proteins in each category (lowest *p*-values) are indicated on the graph. Proteins with the highest fold changes are also labeled. A complete list of the identified proteins can be found in Supplemental Table S1. Dashed vertical lines indicate the fold-change thresholds, and the horizontal dashed line indicates the significance threshold.

**Figure 2:**
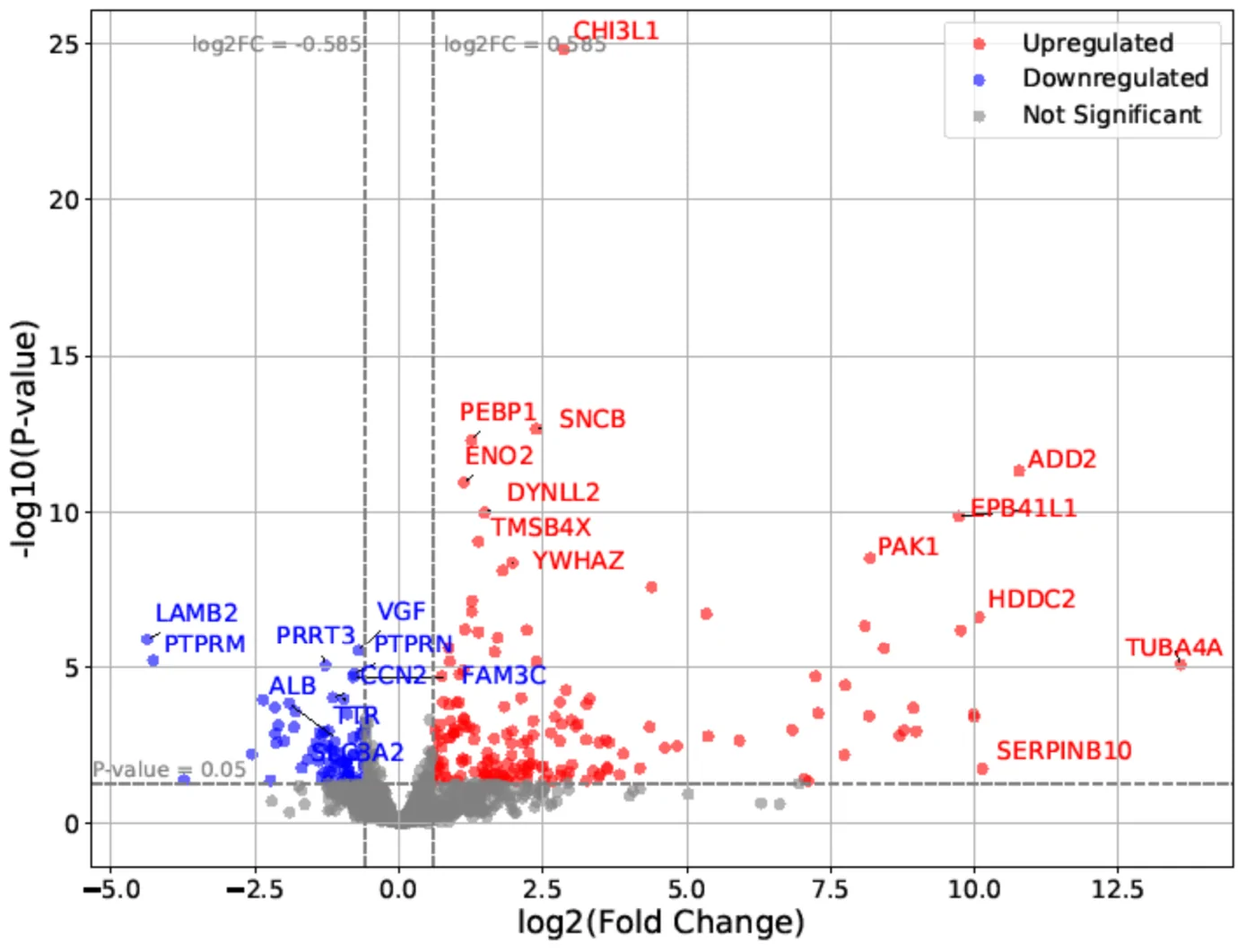
Differentially abundant proteins in CSF from CLN2-affected dogs at the symptomatic time point. Volcano plot showing differential protein abundance between CLN2-affected and age-matched control dogs. The x-axis represents log2 fold change (log2FC), and the y-axis represents −log10(*p*-value). Proteins meeting significance thresholds (raw *p* < 0.05 and log2 fold change >0.585 or <−0.585) are highlighted in red (increased abundance) or blue (decreased abundance), while non-significant proteins are shown in gray. Each dot indicates a specific protein that was identified. Identities of the 10 most significantly altered proteins in each category (lowest *p*-values) are indicated on the graphs (lowest *p*-values). Proteins with the highest fold changes are also labeled. A complete list of the identified proteins can be found in Supplemental Table S2. Dashed vertical lines indicate the fold-change threshold, and the horizontal dashed lines indicate the significance threshold.

**Figure 3:**
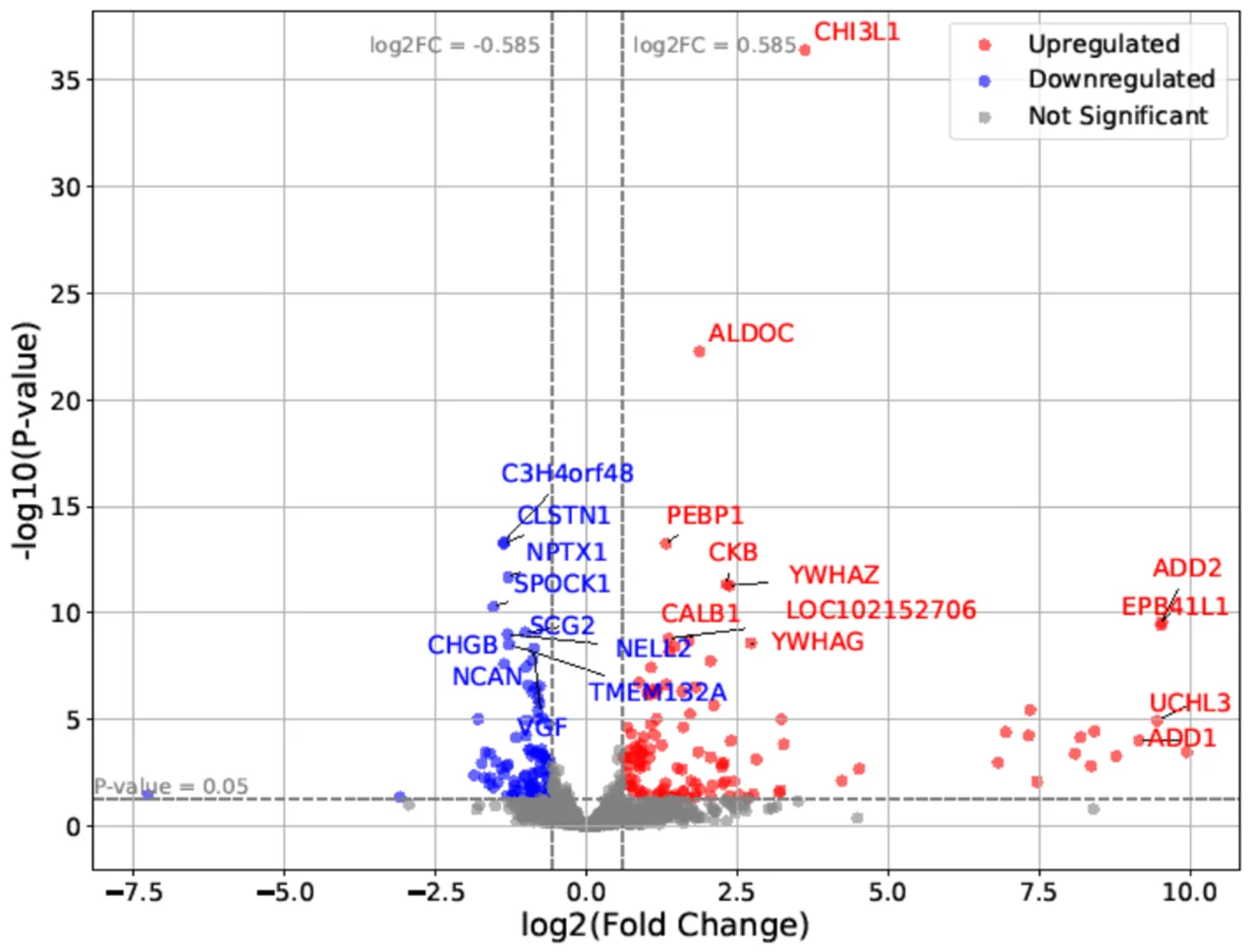
Differentially abundant proteins in CSF from CLN2-affected dogs at the late-stage time point. Volcano plot showing differential protein abundance between CLN2-affected and age-matched control dogs. The x-axis represents log2 fold change (log2FC), and the y-axis represents −log10(*p*-value). Proteins meeting significance thresholds (raw *p* < 0.05 and log2 fold change >0.585 or <−0.585) are highlighted in red (increased abundance) or blue (decreased abundance), while non-significant proteins are shown in gray. Each dot indicates a specific protein that was identified. Identities of the 10 most significantly altered proteins in each category (lowest *p*-values) are indicated on the graphs (lowest *p*-values). Proteins with the highest fold changes are also labeled. A complete list of the identified proteins can be found in Supplemental Table S3. Dashed vertical lines indicate the fold-change threshold, and the horizontal dashed lines indicate the significance threshold.

**Figure 4:**
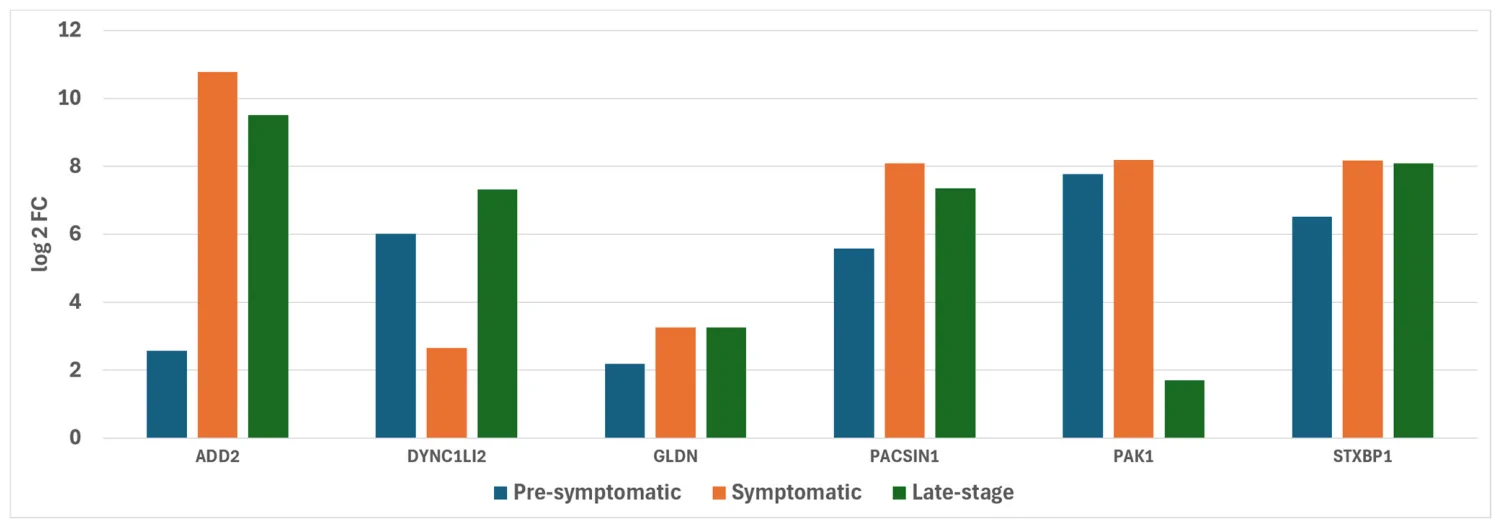
Top 6 differentially altered proteins in CSF across disease stages. Bar plots show log2 fold change (log2FC) values for proteins differentially altered (raw *p*-value < 0.05 and log2 fold change >0.585 or <−0.585) in affected CSF relative to age-matched controls. CSF samples from CLN2-affected dogs exhibited increased abundance of these proteins at all time points evaluated.

1397 proteins were consistently identified in all CSF samples. CSF data for one dog was removed from the CLN2 symptomatic group based on outlier criteria established in Section 2.6. As CLN2-affected dogs progressed from the pre-symptomatic stage to the symptomatic disease stage, there was a notable increase in the number of CSF proteins that were significantly altered when compared to age-matched controls. The presymptomatic, symptomatic, and late-stage time points revealed 69, 263, and 224 significantly altered proteins respectively (Figure 1, Figure 2 and Figure 3 and Supplemental Tables S1–S3). Several proteins were altered in all stages of disease, including adducin 2 [ADD2], dynein light intermediate chain [DYNC1LI2], gliomedin [GLDN], protein kinase C and casein kinase substrate in neurons protein 1 [PACSIN1], non-specific serine/threonine protein kinase [PAK1], and syntaxin-binding protein 1 [STXBP1], all of which showed increased abundance relative to controls (Figure 4). These proteins are involved in cytoskeletal structure, axonal transport, extracellular organization, and synaptic function. In addition, apolipoprotein E [APOE], a protein involved in lipid metabolism and lysosomal function, was significantly elevated at all three time points (presymptomatic: *p* = 0.025, log2FC = 0.71; symptomatic: *p* = 0.002, log2FC = 0.86; late-stage: *p* = 0.001, log2FC = 0.88). The overall persistence of these changes across disease stages indicates that they may be associated with early and ongoing disease-related processes, rather than exclusively reflecting later-stage neuronal degeneration.

At symptomatic and advanced disease stages, a broader set of proteins was differentially abundant (Table 2). These included structural proteins such as neurofilament light [NEFL] and microtubule associated protein tau [MAPT], as well as proteins associated with lysosomal function (e.g., arylsulfatase A, [ARSA], Galectin [LGALSL], transmembrane protein 59 like [TMEM59L]), mitochondrial metabolism (e.g., cytochrome c [CYCS], ATPase inhibitor, mitochondrial [ATP5IF1], creatine kinase B-type [CKB]), and lipid metabolism (e.g., Acyl-CoA-binding protein [DBI], paralemmin 1 [PALM], beta-synuclein [SNCB], triggering receptor expressed on myeloid cells 2 [TREM2]). Additionally, several members of the 14-3-3 protein family (14-3-3 protein epsilon [YWHAE], 14-3-3 protein gamma [YWHAG], 14-3-3 protein theta [YWHAH], and 14-3-3 protein zeta/delta [YWHAZ]) were significantly increased in the CSF at the symptomatic and late-stage time points. These proteins are neuronal adaptor proteins involved in intracellular signaling, cytoskeletal organization, and synaptic function, and their elevation in CSF has been associated with neuronal injury and neurodegeneration [41,42].

**Figure 5:**
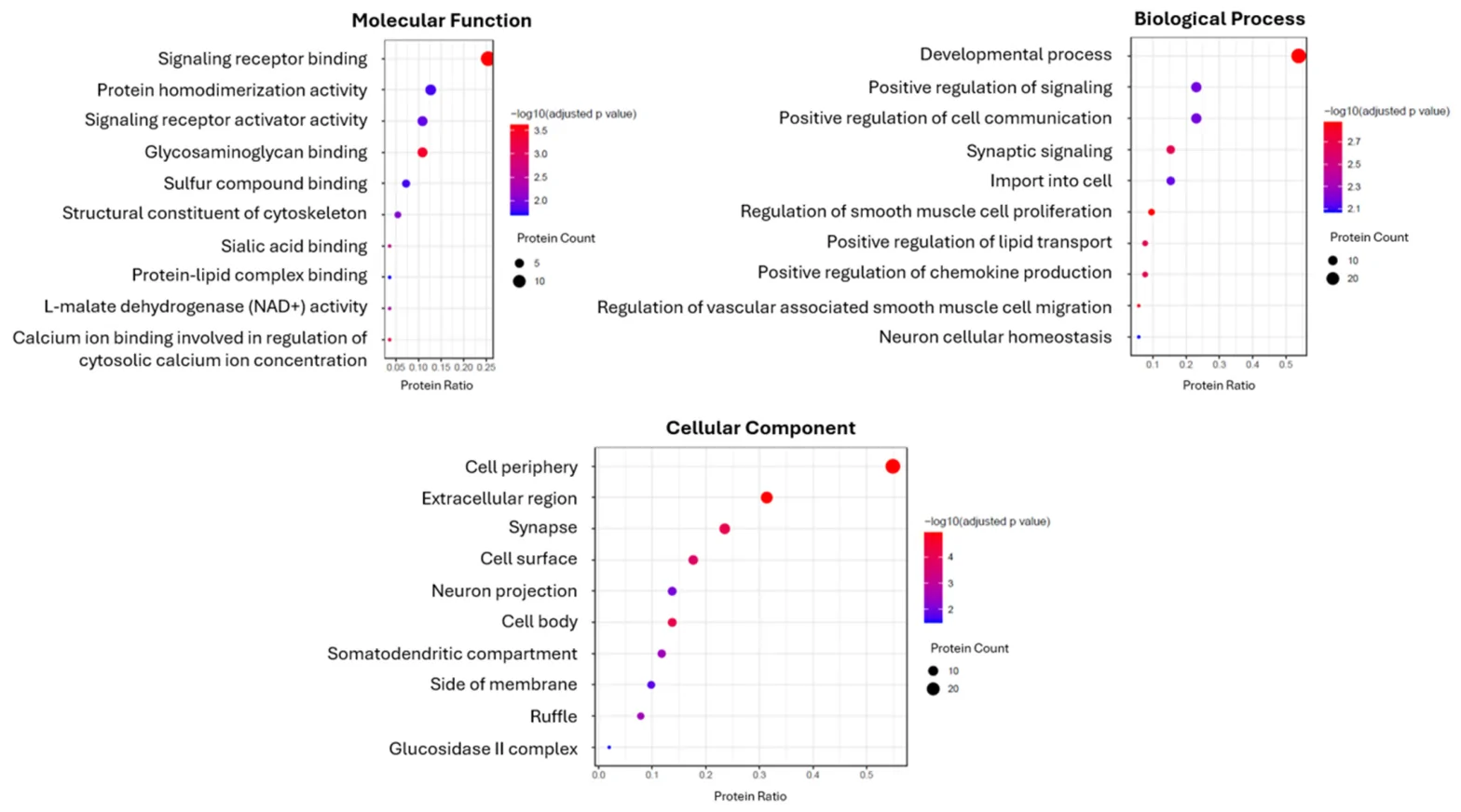
Gene ontology (GO) enrichment analysis of differentially abundant CSF proteins at the presymptomatic time point in CLN2-affected dogs. Dot plots showing the top 10 enriched GO terms for molecular function (**top left**), biological process (**top right**), and cellular component (**bottom**) categories. The x-axis represents gene ratio (proportion of differentially abundant proteins associated with a given term), while dot size indicates the number of proteins assigned to each term. Dot color corresponds to the significance of enrichment, expressed as −log10 (adjusted *p*-value), with warmer colors indicating greater statistical significance.

**Figure 6:**
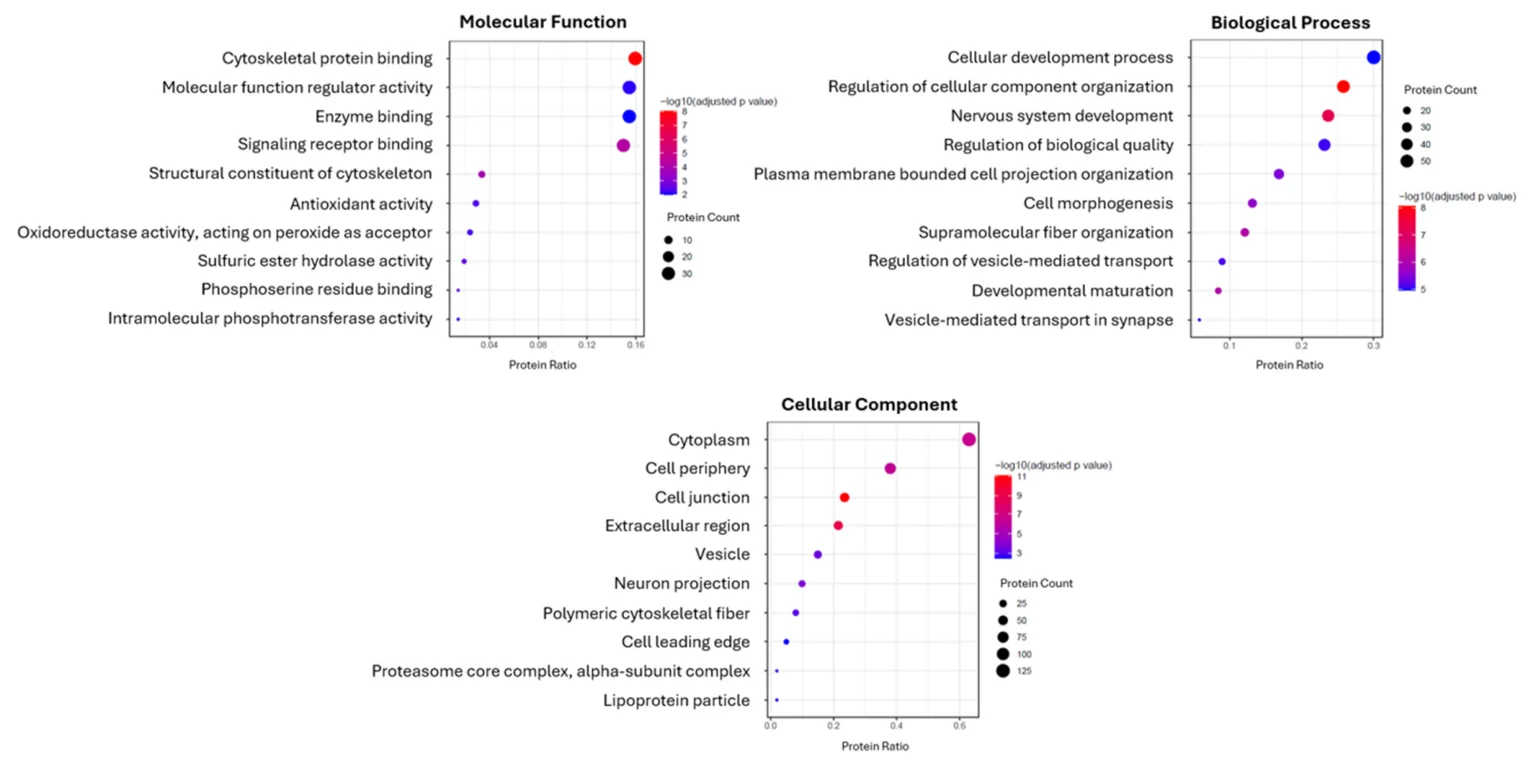
Gene ontology (GO) enrichment analysis of differentially abundant CSF proteins at symptomatic time point in CLN2-affected dogs. Dot plots showing the top 10 enriched GO terms for molecular function (**top left**), biological process (**top right**), and cellular component (**bottom**) categories. The x-axis represents gene ratio (proportion of differentially abundant proteins associated with a given term), while dot size indicates the number of proteins assigned to each term. Dot color corresponds to the significance of enrichment, expressed as −log10 (adjusted *p*-value), with warmer colors indicating greater statistical significance.

**Figure 7:**
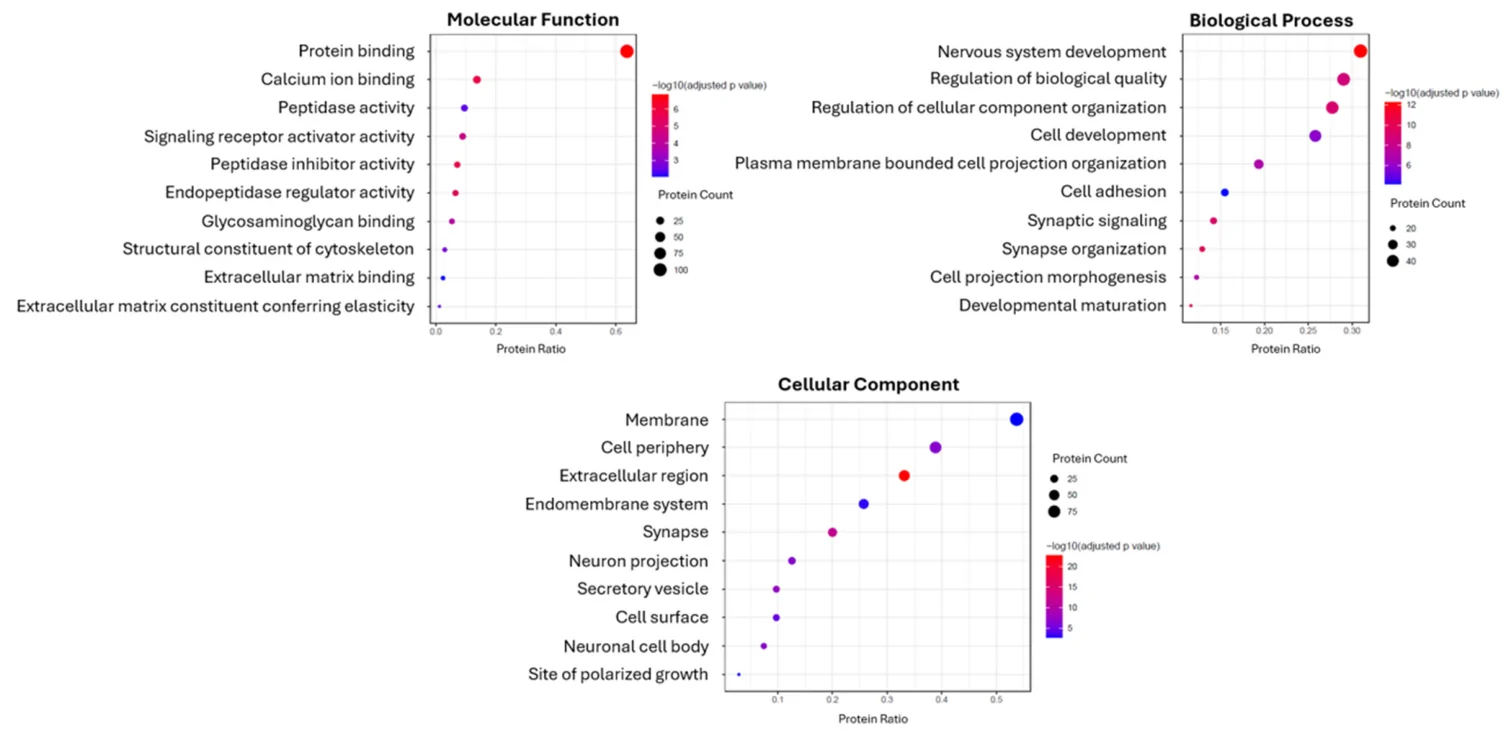
Gene ontology (GO) enrichment analysis of differentially abundant CSF proteins at the late-stage time point in CLN2-affected dogs. Dot plots showing the top 10 enriched GO terms for molecular function (**top left**), biological process (**top right**), and cellular component (**bottom**) categories. The x-axis represents gene ratio (proportion of differentially abundant proteins associated with a given term), while dot size indicates the number of proteins assigned to each term. Dot color corresponds to the significance of enrichment, expressed as −log10 (adjusted *p*-value), with warmer colors indicating greater statistical significance.

Gene ontology (GO) analysis of CSF proteins identified recurring enrichment of pathways associated with neuronal development and signaling, cellular organization, cytoskeletal structure, vesicle-mediated transport, and immune/inflammatory responses across disease progression (Figure 5, Figure 6 and Figure 7 and Supplemental Tables S4–S6). At the presymptomatic time point, the most significantly enriched biological processes included development, regulation of lipid transport, and synaptic signaling, while extracellular region and synapse were the predominant enriched cellular components. Molecular function analysis highlighted receptor binding, calcium ion binding, structural constituent of cytoskeleton, and protein-lipid complex binding.

At the symptomatic time point, enrichment patterns expanded considerably and were dominated by pathways related to regulation of cellular component organization and nervous system development. Additional enriched terms included vesicle-mediated transport, cytoskeletal organization, synaptic signaling, signal release, and immune/inflammatory responses. Enriched cellular components included cell junctions, extracellular regions, vesicles, polymetric cytoskeletal fibers, and cytochrome complexes, while molecular functions were strongly associated with cytoskeletal protein binding, signaling receptor binding, and structural constituent of cytoskeleton. These findings are consistent with progressive disruption of neuronal structural integrity, intracellular trafficking, and inflammatory signaling during disease progression.

At the late-stage time point, the most significantly enriched pathways included nervous system development, synapse organization, developmental maturation, and synaptic signaling. Additional enrichment of pathways related to vesicle-mediated transport, protein localization to synapses, signal release, and cytoskeletal organization further supported widespread disruption of neuronal communication and synaptic function during advanced disease. Synapse and secretory vesicle were the most significantly enriched cellular components, while molecular function analysis identified enrichment of protein binding, peptidase inhibitor activity, calcium ion binding, lipid binding, and structural constituent of cytoskeleton.

Collectively, these findings demonstrate progressive alterations in the CSF proteome throughout the disease course, with distinct and overlapping sets of significantly altered proteins across presymptomatic, symptomatic, and advanced disease stages. The enrichment pathways span multiple functional categories, supporting coordinated disruption of neuronal structures, synaptic organization, intracellular transport, and inflammatory-associated processes over the course of disease progression.

### 3.3. Plasma Protein Alterations

**Figure 8:**
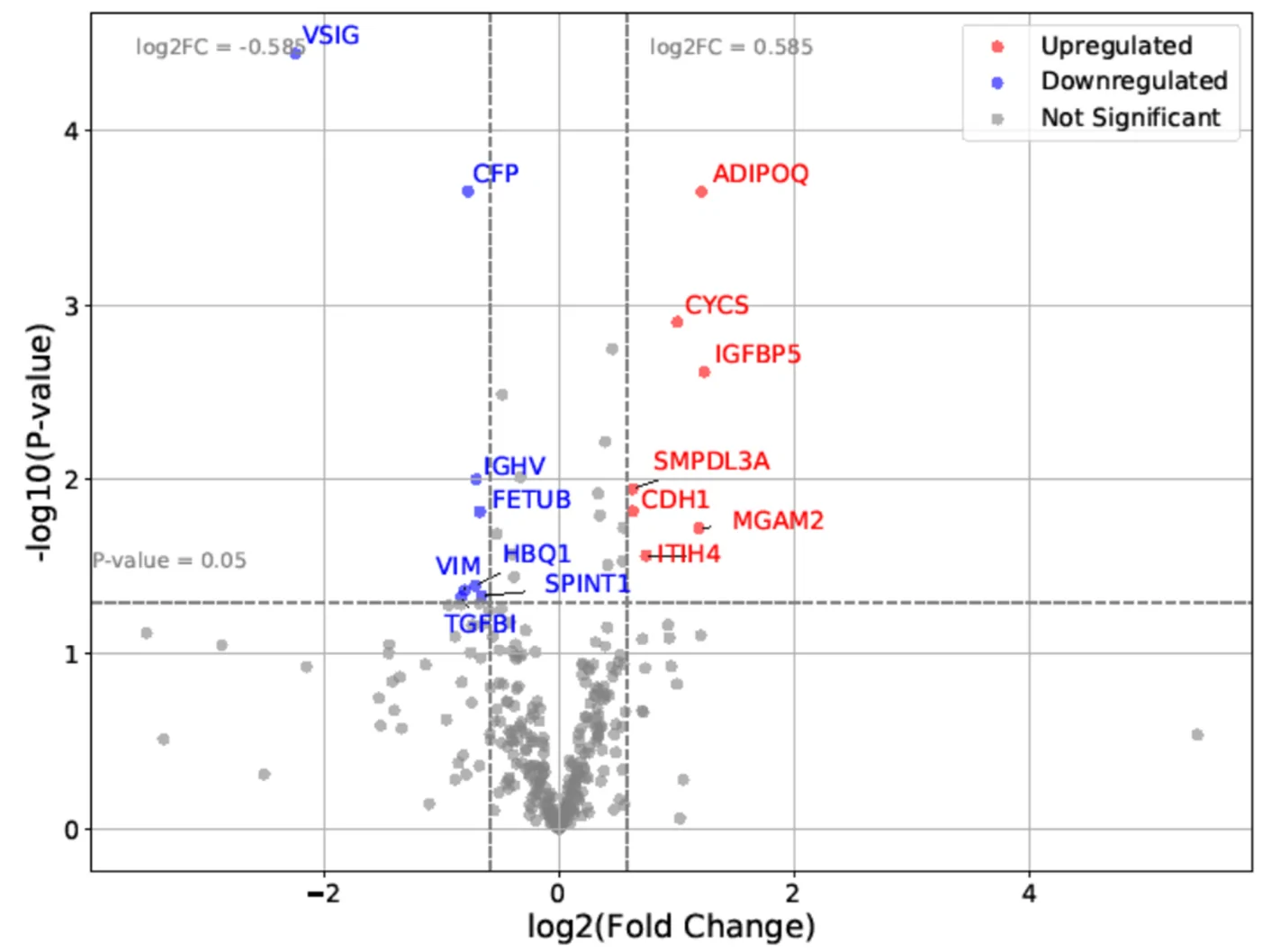
Differentially abundant proteins in plasma from CLN2-affected dogs at the presymptomatic time point. Volcano plot showing differential protein abundance between CLN2-affected and age-matched control dogs. The x-axis represents log2 fold change (log2FC), and the y-axis represents −log10(*p*-value). Proteins meeting significance thresholds (raw *p* < 0.05 and log2 fold change >0.585 or <−0.585) are highlighted in red (increased abundance) or blue (decreased abundance), while non-significant proteins are shown in gray. Each dot indicates a specific protein that was identified. Identities of the 10 most significantly altered proteins in each category are indicated on the graphs (lowest *p*-values). A complete list of the identified proteins can be found in Table 3. Dashed vertical lines indicate the fold-change threshold, and the horizontal dashed lines indicate the significance threshold.

**Figure 9:**
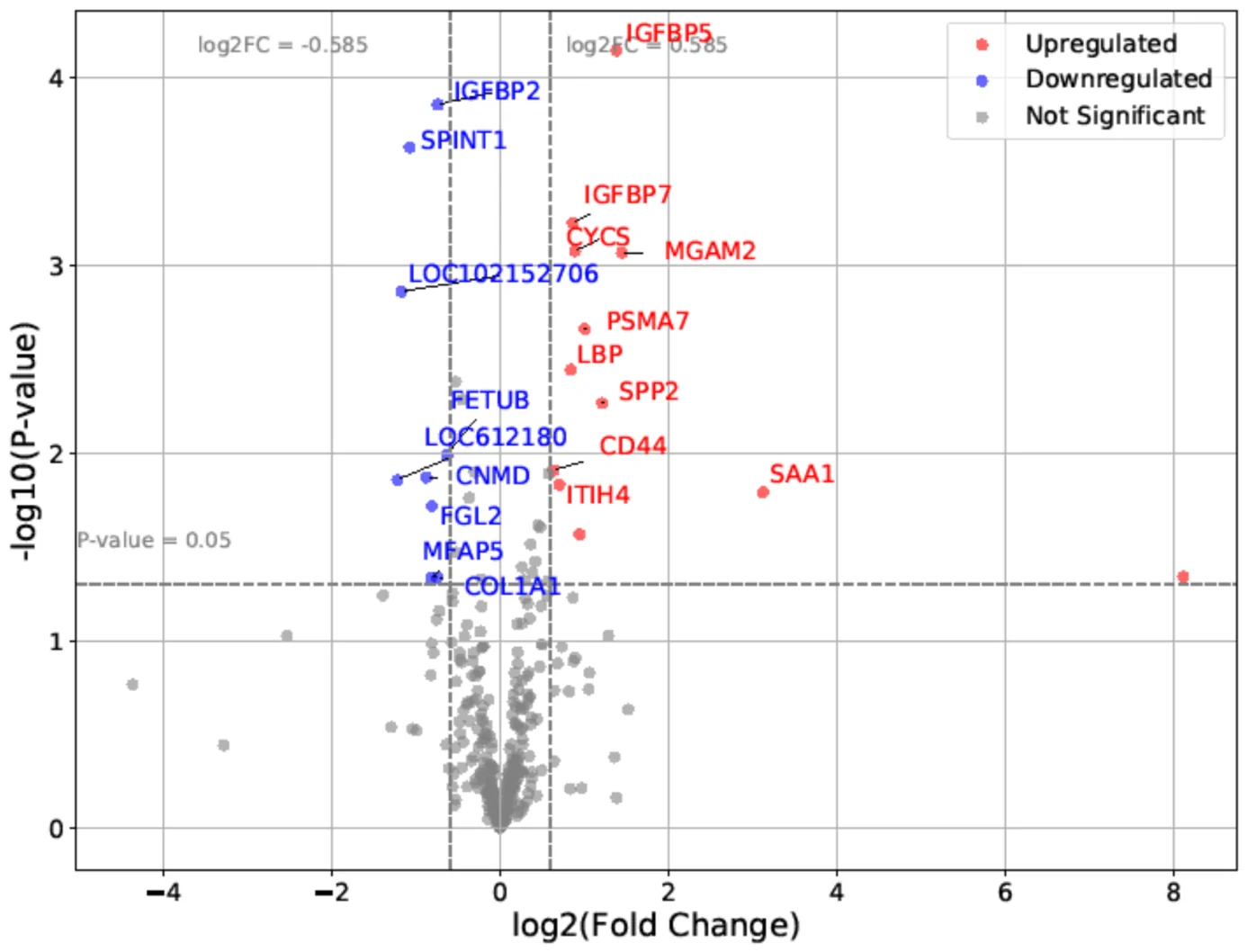
Differentially abundant proteins in plasma from CLN2-affected dogs at symptomatic time point. Volcano plot showing differential protein abundance between CLN2-affected and age-matched control dogs. The x-axis represents log2 fold change (log2FC), and the y-axis represents −log10(*p*-value). Proteins meeting significance thresholds (raw *p* < 0.05 and log2 fold change >0.585 or <−0.585) are highlighted in red (increased abundance) or blue (decreased abundance), while non-significant proteins are shown in gray. Each dot indicates a specific protein that was identified. Identities of the 10 most significantly altered proteins in each category are indicated on the graphs (lowest *p*-values). A complete list of the identified proteins can be found in Table 3. Dashed vertical lines indicate the fold-change threshold, and the horizontal dashed lines indicate the significance threshold.

**Figure 10:**
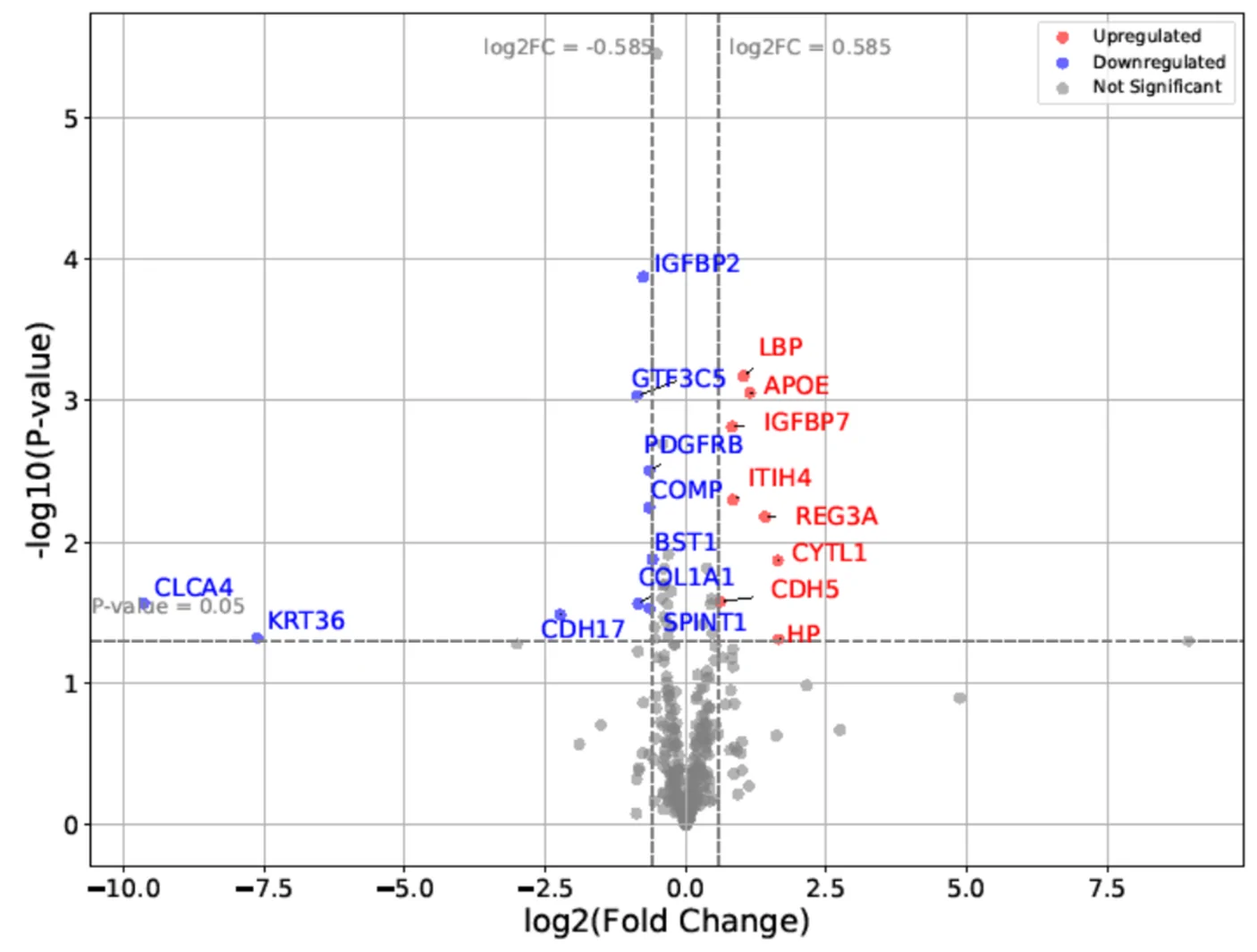
Differentially abundant proteins in plasma from CLN2-affected dogs at the late-stage time point. Volcano plot showing differential protein abundance between CLN2-affected and age-matched control dogs. The x-axis represents log2 fold change (log2FC), and the y-axis represents −log10(*p*-value). Proteins meeting significance thresholds (raw *p* < 0.05 and log2 fold change >0.585 or <−0.585) are highlighted in red (increased abundance) or blue (decreased abundance), while non-significant proteins are shown in gray. Each dot indicates a specific protein that was identified. Identities of the 10 most significantly altered proteins in each category are indicated on the graphs (lowest *p*-values). A complete list of the identified proteins can be found in Table 3. Dashed vertical lines indicate the fold-change threshold, and the horizontal dashed lines indicate the significance threshold.

A total of 331 proteins were identified in plasma. Of these, 21, 21, and 22 proteins were significantly altered at the presymptomatic, symptomatic, and late-stage time points, respectively (Figure 8, Figure 9 and Figure 10 and Table 3). In contrast to CSF, relatively few proteins overlapped across time points, and those that did generally exhibited modest fold changes. Cytochrome c [CYCS] was among the few proteins consistently elevated, reaching statistical significance at the presymptomatic time point (raw *p* = 0.001, log2FC = 1.01) and symptomatic time point (raw *p* = 0.001, log2FC = 0.89). A similar trend was observed at late-stage, but this change was not statistically significant.

**Figure 11:**
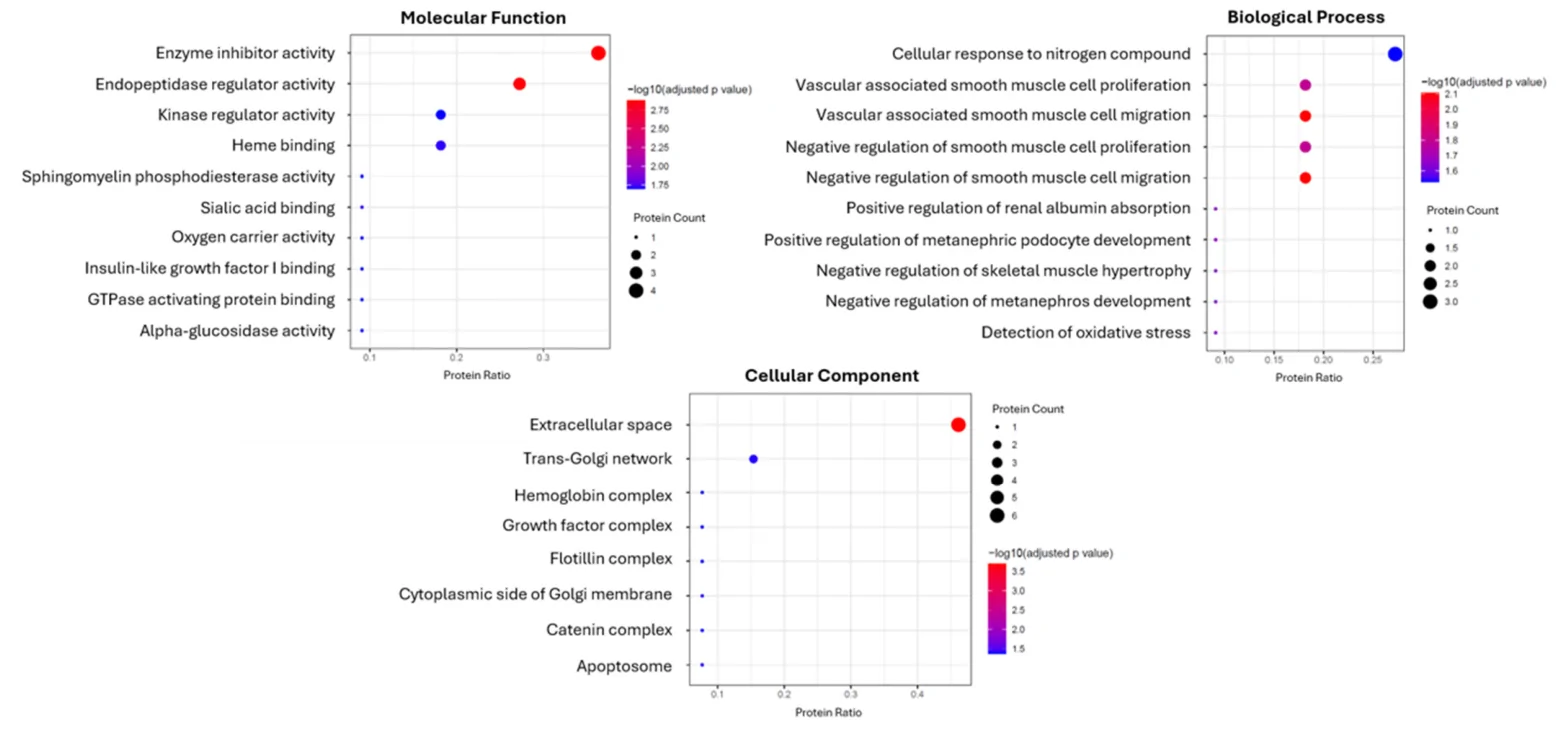
Gene ontology (GO) enrichment analysis of differentially abundant plasma proteins at the presymptomatic time point in CLN2-affected dogs. Dot plots showing the top enriched GO terms for molecular function (**top left**), biological process (**top right**), and cellular component (**bottom**) categories. The x-axis represents gene ratio (proportion of differentially abundant proteins associated with a given term), while dot size indicates the number of proteins assigned to each term. Dot color corresponds to the significance of enrichment, expressed as −log10 (adjusted *p*-value), with warmer colors indicating greater statistical significance.

**Figure 12:**
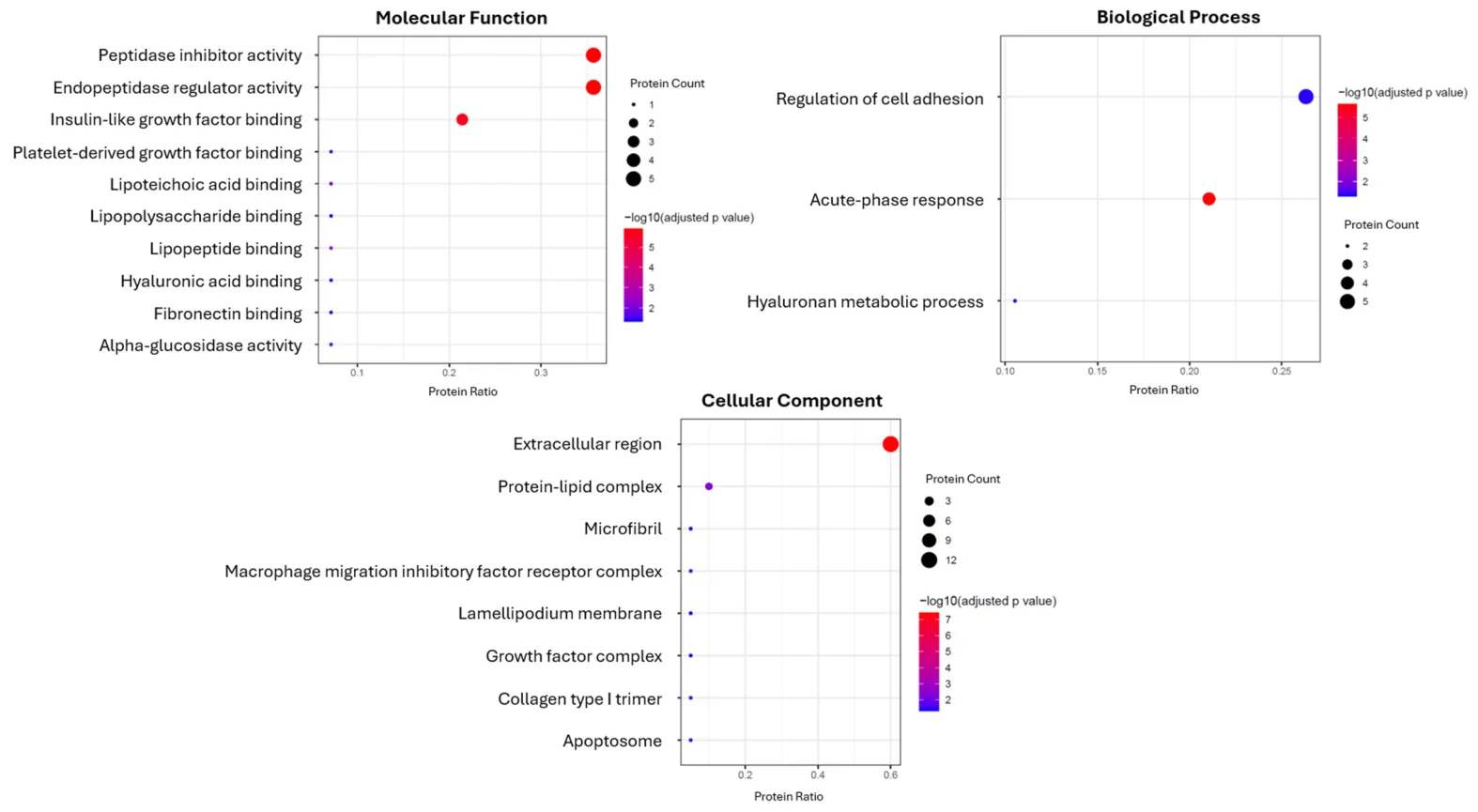
Gene ontology (GO) enrichment analysis of differentially abundant plasma proteins at the symptomatic time point in CLN2-affected dogs. Dot plots showing the top enriched GO terms for molecular function (**top left**), biological process (**top right**), and cellular component (**bottom**) categories. The x-axis represents gene ratio (proportion of differentially abundant proteins associated with a given term), while dot size indicates the number of proteins assigned to each term. Dot color corresponds to the significance of enrichment, expressed as −log10 (adjusted *p*-value), with warmer colors indicating greater statistical significance.

**Figure 13:**
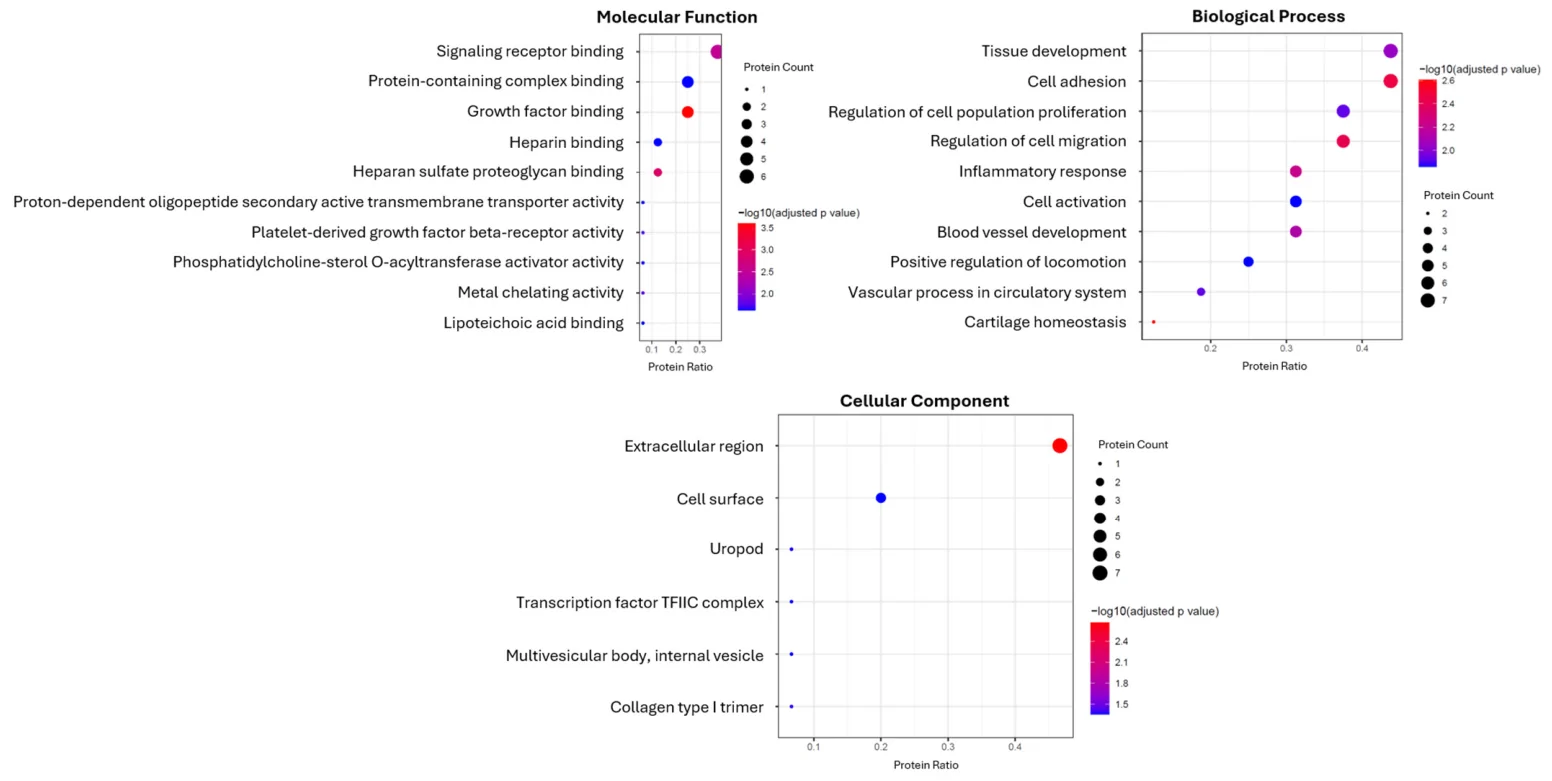
Gene ontology (GO) enrichment analysis of differentially abundant plasma proteins at the late-stage time point in CLN2-affected dogs. Dot plots showing the top enriched GO terms for molecular function (**top left**), biological process (**top right**), and cellular component (**bottom**) categories. The x-axis represents gene ratio (proportion of differentially abundant proteins associated with a given term), while dot size indicates the number of proteins assigned to each term. Dot color corresponds to the significance of enrichment, expressed as −log10 (adjusted *p*-value), with warmer colors indicating greater statistical significance.

Gene ontology (GO) analysis of plasma proteins is summarized in Figure 11, Figure 12 and Figure 13 and Supplemental Tables S7–S9. Compared to CSF, plasma demonstrated fewer enriched pathways and less consistent enrichment across disease stages. Across all time points, recurring themes included altered extracellular and cell adhesion-associated processes, with the extracellular region/space representing the most consistently enriched cellular compartment. Molecular function analysis also repeatedly identified enrichment of enzyme and peptidase inhibitor activity, suggesting persistent alterations in extracellular signaling and regulatory processes throughout disease progression. At the presymptomatic time point, the most significantly enriched pathways were associated with smooth muscle cell migration and regulation of cell–cell adhesion, and at the symptomatic time point, acute-phase responses and regulation of cell adhesion represented the predominant biological categories. At late-stage, enrichment patterns expanded to include pathways related to inflammatory response, extracellular structure organization, lipid transport, cholesterol metabolism, and fatty acid homeostasis, in addition to continued enrichment of cell adhesion-associated processes. Molecular function analysis at this later disease stage also identified enrichment of growth factor binding, signaling receptor binding, calcium ion binding, and cholesterol transfer activity. Overall, plasma GO analysis identified modest but recurring enrichment of extracellular, inflammatory, and lipid-associated pathways, further highlighting the systemic alterations of signaling, immune response, and lipid metabolism in CLN2 disease progression.

### 3.4. Tear Fluid Protein Alterations

**Figure 14:**
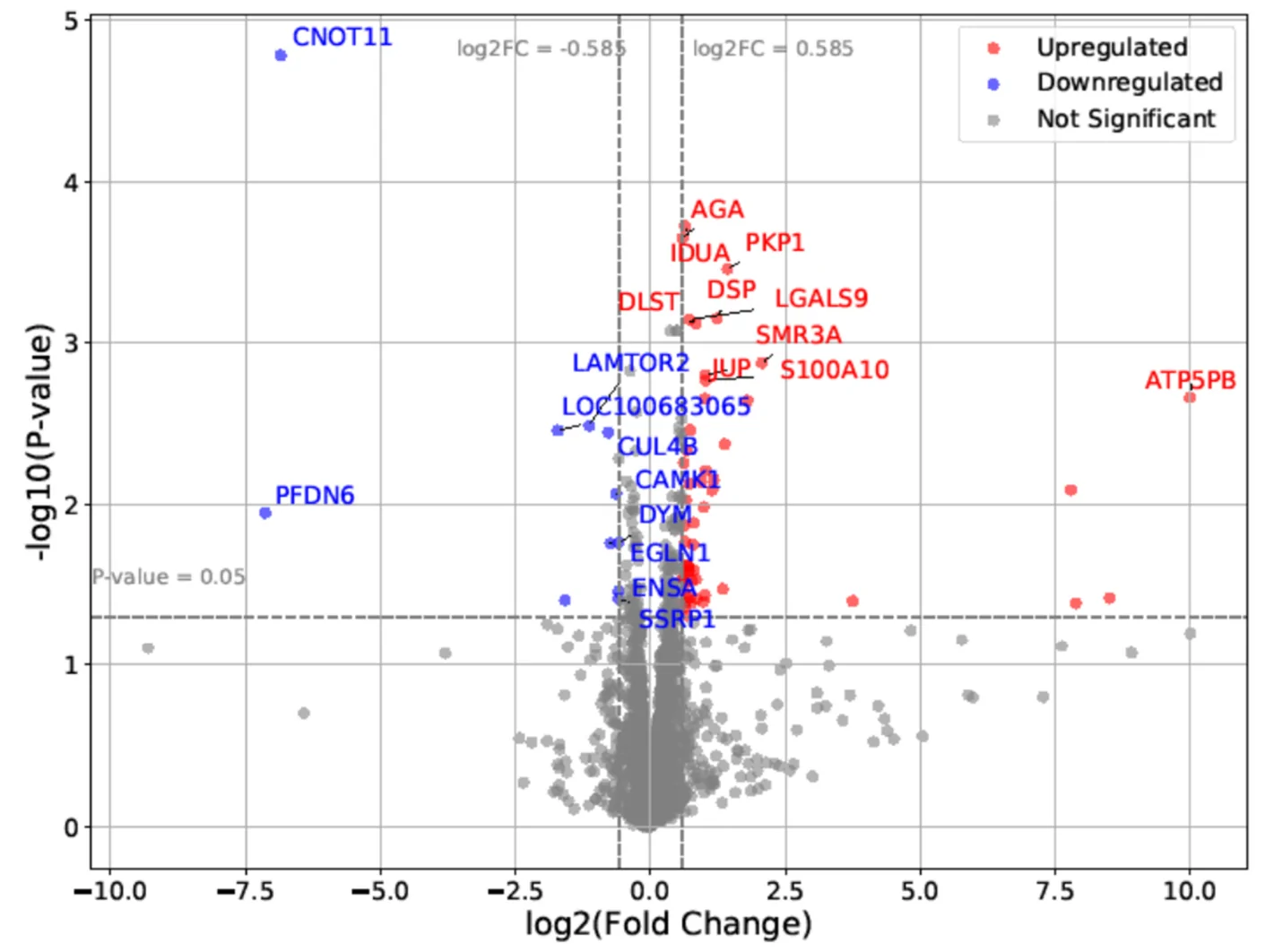
Differentially abundant proteins in tears from CLN2-affected dogs at the presymptomatic time point. Volcano plot showing differential protein abundance between CLN2-affected and age-matched control dogs. The x-axis represents log2 fold change (log2FC), and the y-axis represents −log10(*p*-value). Proteins meeting significance thresholds (raw *p* < 0.05 and log2 fold change >0.585 or <−0.585) are highlighted in red (increased abundance) or blue (decreased abundance), while non-significant proteins are shown in gray. Each dot indicates a specific protein that was identified. Identities of the 10 most significantly altered proteins in each category are indicated on the graphs (lowest *p*-values). A complete list of the identified proteins can be found in Supplemental Table S12. Dashed vertical lines indicate the fold-change threshold, and the horizontal dashed lines indicate the significance threshold.

**Figure 15:**
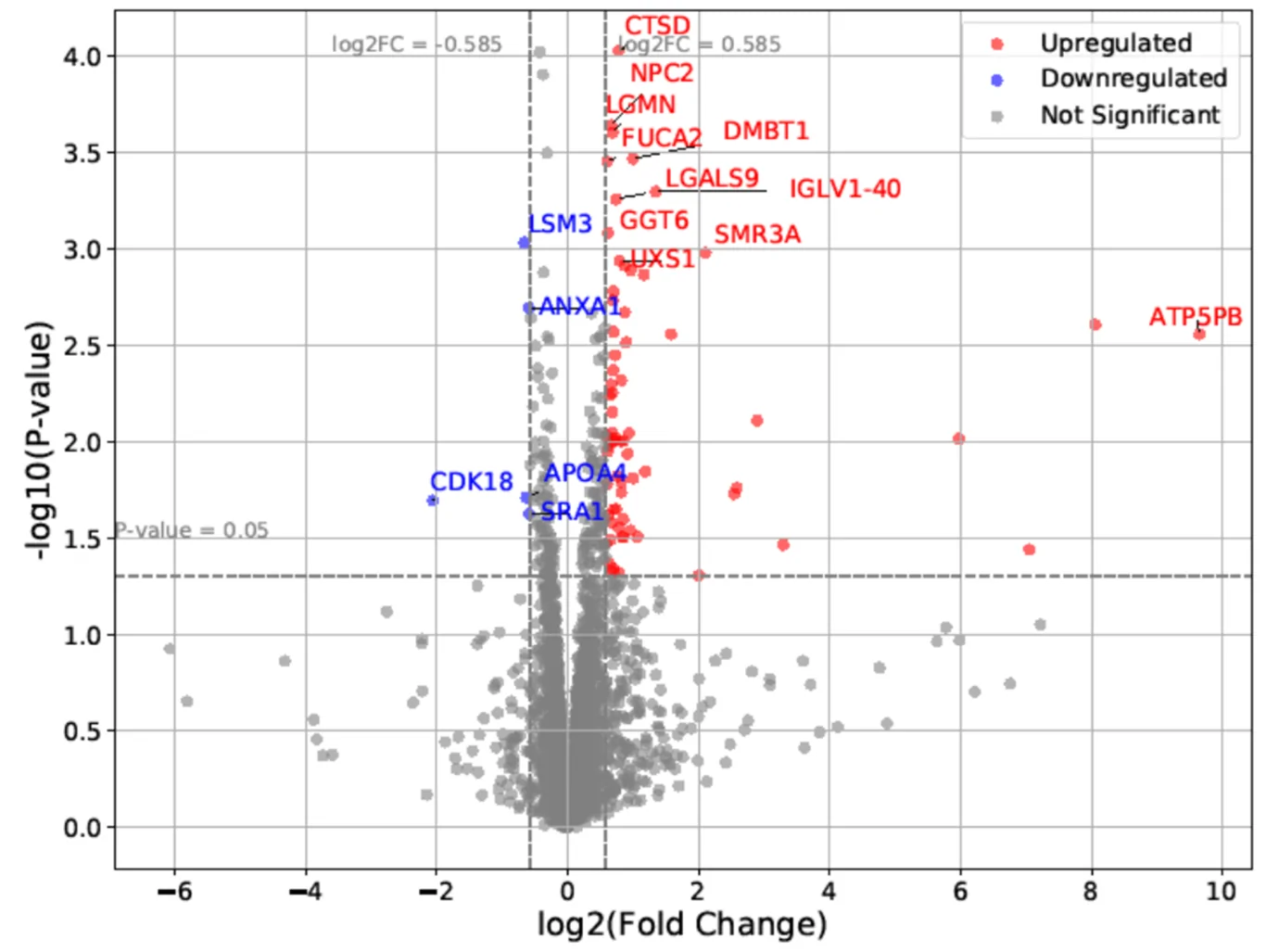
Differentially abundant proteins in tears from CLN2-affected dogs at symptomatic time point. Volcano plot showing differential protein abundance between CLN2-affected and age-matched control dogs. The x-axis represents log2 fold change (log2FC), and the y-axis represents −log10(*p*-value). Proteins meeting significance thresholds (raw *p* < 0.05 and log2 fold change >0.585 or <−0.585) are highlighted in red (increased abundance) or blue (decreased abundance), while non-significant proteins are shown in gray. Each dot indicates a specific protein that was identified. Identities of the 10 most significantly altered proteins in each category are indicated on the graphs (lowest *p*-values). A complete list of the identified proteins can be found in Supplemental Table S13. Dashed vertical lines indicate the fold-change threshold, and the horizontal dashed lines indicate the significance threshold.

**Figure 16:**
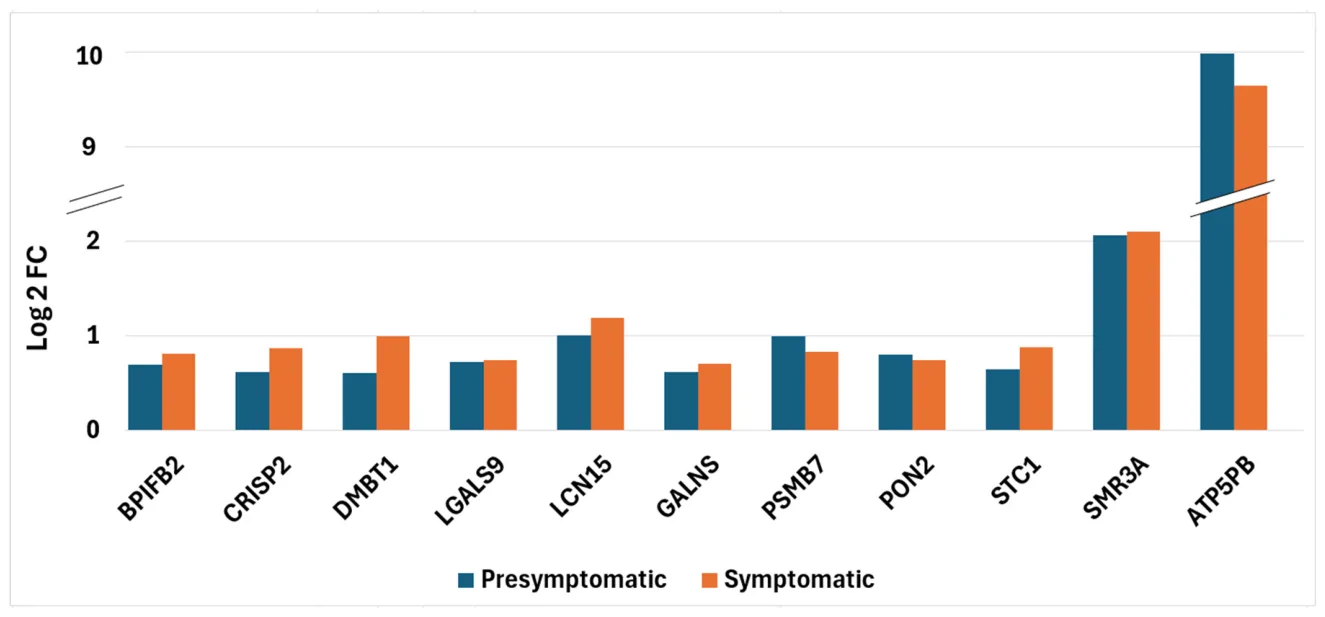
Differentially altered proteins in tear fluid at presymptomatic and early symptomatic stages. Bar plots show log2 fold change (log2FC) of significantly changed proteins identified in tear fluid at the presymptomatic (blue) and symptomatic (orange) stages relative to age-matched controls. Protein abundance was significantly altered in affected samples at both time points, highlighting early and persistent changes detected in the tear proteome during disease progression.

A total of 3705 proteins were identified in tears; 68 and 87 proteins were significantly altered in samples from CLN2-affected dogs at the presymptomatic and symptomatic time points, respectively (Figure 14 and Figure 15 and Supplemental Tables S10 and S11). A subset of proteins was modestly elevated at both time points analyzed (Figure 16), revealing biological themes that parallel those observed in CSF. Proteins associated with lysosomal function were represented, including galectin 9 [LGALS9] and N-acetylgalactosamine-6-sulfatase [GALNS], which were elevated at both time points. In addition, several proteins related to immune and inflammatory processes were increased, including BPI fold-containing family B, member 2 [BPIFB2], deleted in malignant brain tumors 1 [DMBT1], and submaxillary gland androgen-regulated protein 3A [SMR3A]. Proteins associated with lipid metabolism and transport were also elevated, including lipocalin/cytosolic fatty-acid binding domain-containing protein [LCN15] and serum paraoxonase/arylesterase 2 [PON2]. Additionally, proteins linked to mitochondrial metabolism and cellular stress response were increased (e.g., stanniocalcin 1 [STC1] and ATP synthase F (0) complex subunit B1, mitochondrial [ATP5PB]). ATP5PB showed the largest magnitude change at both time points and was undetectable in the tears of unaffected control dogs.

**Figure 17:**
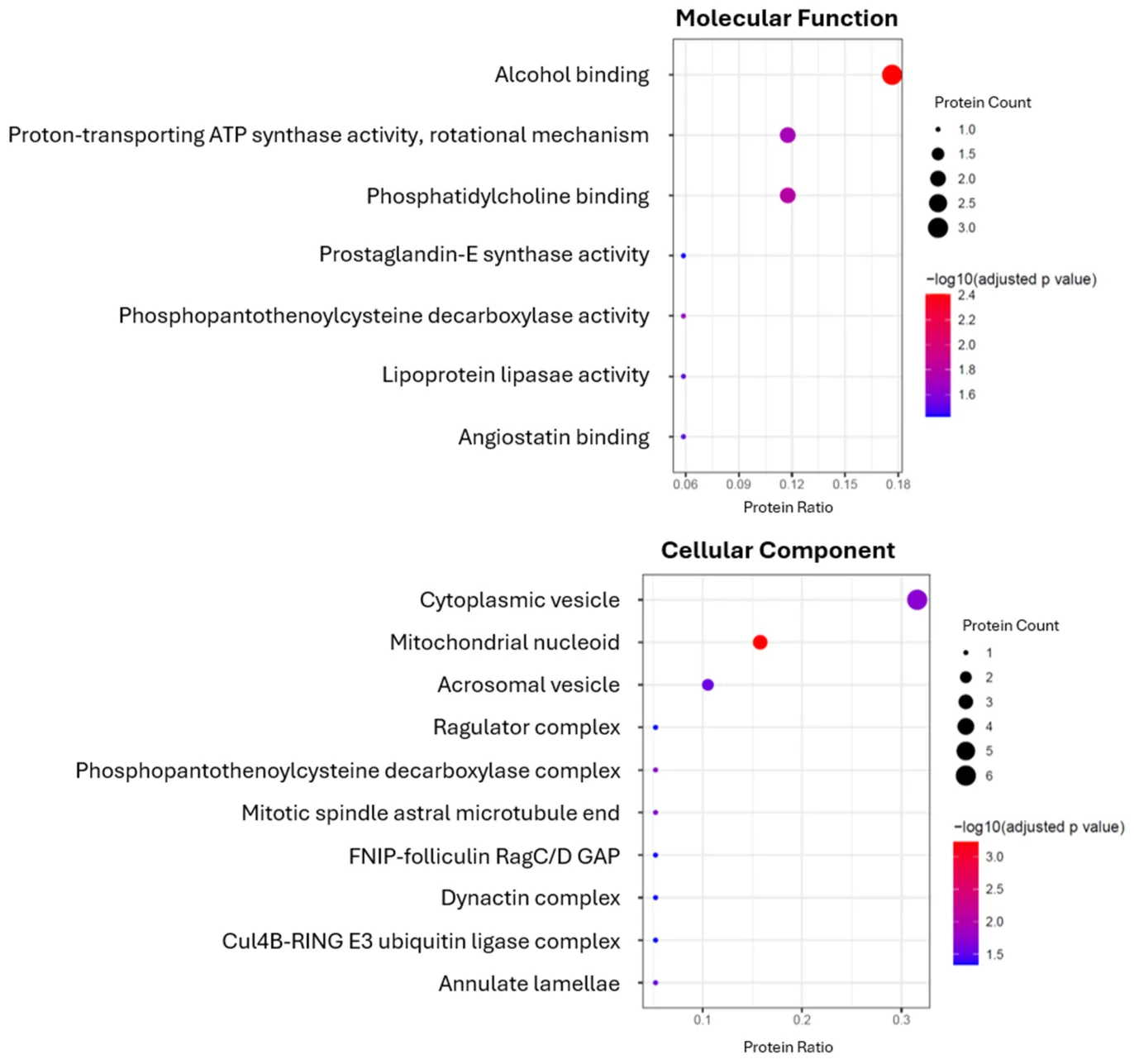
Gene ontology (GO) enrichment analysis of differentially abundant tear proteins at the presymptomatic time point in CLN2-affected dogs. Dot plots showing the top enriched GO terms for molecular function (**top**) and cellular component (**bottom**) categories. The x-axis represents gene ratio (proportion of differentially abundant proteins associated with a given term), while dot size indicates the number of proteins assigned to each term. Dot color corresponds to the significance of enrichment, expressed as −log10 (adjusted *p*-value), with warmer colors indicating greater statistical significance.

**Figure 18:**
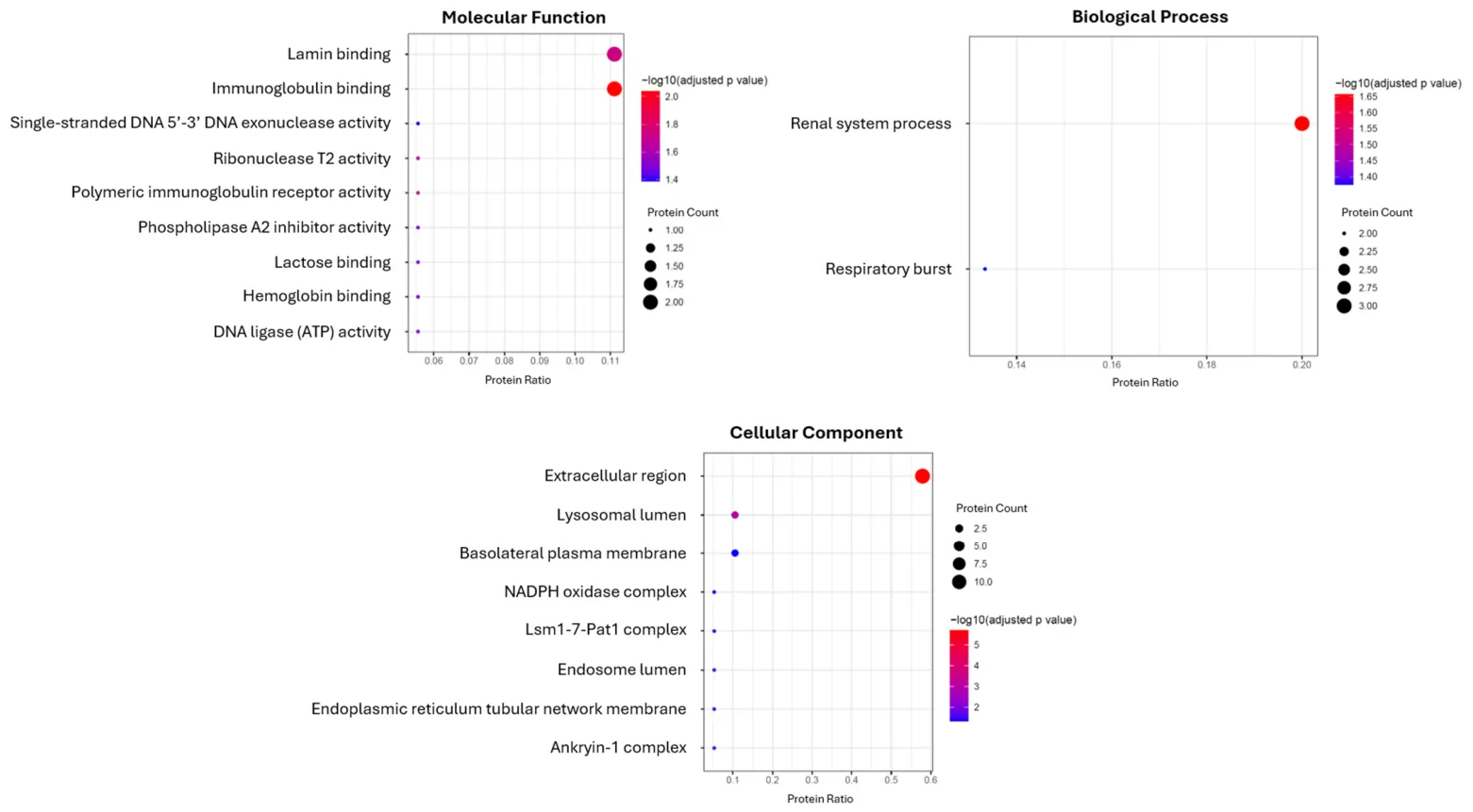
Gene ontology (GO) enrichment analysis of differentially abundant tear proteins at the symptomatic time point in CLN2-affected dogs. Dot plots showing the top enriched GO terms for molecular function (**top**) and cellular component (**bottom**) categories. The x-axis represents gene ratio (proportion of differentially abundant proteins associated with a given term), while dot size indicates the number of proteins assigned to each term. Dot color corresponds to the significance of enrichment, expressed as −log10 (adjusted *p*-value), with warmer colors indicating greater statistical significance.

Gene ontology (GO) analysis is summarized in Figure 17 and Figure 18 and Supplemental Tables S12 and S13. At the presymptomatic time point, the mitochondrial matrix and proton-transporting ATP synthase complex, catalytic core F (1), were significantly enriched cellular compartments. Mitochondrial proteins were also increased in affected tears, including ATP synthase F1 subunits alpha [ATP5F1A] and beta [ATP5F1B], trifunctional enzyme subunit alpha, mitochondrial [HADHA], voltage-dependent ion channel 2 [VDAC2], NADH dehydrogenase [ubiquinone] 1 alpha subcomplex subunit 9 [NDUFA9], ADP/ATP translocase 2 [SLC25A5], and acetyl-CoA acetyltransferase, mitochondrial [ACAT1]. These proteins are primarily involved in such activities as mitochondrial energy production, electron transport chain activity, ATP synthesis and transport, and fatty acid β-oxidation.

At the symptomatic time point, key driver terms in the GO analysis included amyloid fibril formation, amyloid-beta clearance, and lipid transport. The lysosomal lumen was the second most overrepresented cellular compartment, with the proteins N-sulfoglucosamine sulfohydrolase [SGSH], ribonuclease T2 [RNASET2], and phospholipase D family member 3 [PLD3] increased in affected dogs. While not identified by GO analysis, other lysosomal lumen proteins, including the lysosomal hydrolases cathepsin D [CTSD] and cathepsin C [CTSC], as well as the lysosomal membrane protein NPC intracellular cholesterol transporter 2 [NPC2], were elevated in affected dogs, demonstrating that compensatory elevations in other lysosomal proteins in CLN2 disease can be detected in tear fluid at later disease stages.

### 3.5. Cross-Biofluid Correlation Analysis

Classification performance of the DIABLO model was evaluated using Leave-One-Animal-Out cross-validation, yielding a balanced error rate of 4.5% (10 of 11 Affected and 9 of 9 Control samples correctly classified).

Pairwise correlation analysis was performed to identify coordinated protein abundance patterns across biofluids, and the resulting associations were visualized using a circos plot and correlation heatmap (Supplemental Table S14 and Supplemental Figures S1 and S2). After filtering for strong correlations (|r| ≥ 0.7), a total of 7520 pairwise associations were identified between proteins measured in tear fluid, CSF, and plasma, including both positive and negative relationships. Proteins involved in cytoskeletal organization and axonal structure were prominently represented among correlated pairs. Proteins associated with synaptic function, vesicle trafficking, and neuronal signaling were also frequently observed. In addition, microtubule motor and transport proteins involved in axonal transport were recurrently represented (Table 4). Additional noted groups of correlated pairs were associated with lysosomal biology, proteostasis, and cellular stress responses. Several of these proteins appeared in repeated correlations between tear fluid and CSF, aligning with their differential abundance levels at later disease stages. Proteins involved in lipid metabolism and transport were also represented. APOE appeared in multiple correlations spanning CSF and plasma. Immune and inflammatory proteins were similarly enriched among correlated pairs (Table 4).

Associations were also observed between CSF neurofilament light [NEFL] levels and proteins detected in the other biofluids, particularly tears (Supplemental Table S14). The highest positive correlations were observed between NEFL in CSF and tear proteins angiotensin-converting enzyme 2 [ACE2], melanotransferrin [MELTF], ribonuclease T2 [RNASET2], N-sulfoglucosamine sulfohydrolase [SGSH], legumain [LGMN], and aarboxypeptidase Q [CPQ], along with additional lysosomal and glycosaminoglycan-related proteins N-acetylgalactosamine-6-sulfatase [GALNS], NPC intracellular cholesterol transporter 2 [NPC2], cathepsin D [CTSD], Alpha-L-iduronidase [IDUA], and alpha-L-fucosidase [FUCA2]. Proteins involved in immune and barrier function (JCHAIN, PIGR, CLU) and metabolic regulation (PON3, IGFBP7) were also positively correlated with CSF NEFL. In contrast, fewer plasma proteins showed positive associations with NEFL, including adiponectin [ADIPOQ] and insulin-like growth factor-binding protein 5 [IGFBP5], while several plasma proteins (vimentin [VIM], chondromodulin-1 [CNMD], microfibrillar-associated protein 5 [MFAP5], and collagen alpha-1 (I) chain [COL1A1]) displayed negative correlations with NEFL.

While primarily exploratory/descriptive in nature, these results suggest that proteins exhibiting differential abundance across disease stages also exhibit cross-biofluid relationships. These associations were most prominent between CSF and tear fluid, with fewer involving plasma, consistent with patterns observed in differential abundance and pathway enrichment analysis. Together, the data support the presence of shared molecular features across biofluids, particularly involving cytoskeletal, synaptic, lysosomal, lipid metabolism, mitochondrial, and immune-related processes.

## 4. Discussion

This study provides the first proteomic characterization of CSF, tear fluid, and plasma in a large animal model of CLN2 disease and identifies molecular alterations across multiple biofluids over the course of the disease. A major feature of the dataset was the apparent increase in both the number and magnitude of altered proteins at symptomatic and advanced disease stages, compared to the presymptomatic stage, consistent with previous CLN2 proteomic studies [18]. Unequal and small group size across biofluids and disease stages likely reduced statistical power in several comparisons, yielding less stable dispersion and fold-change estimates and potentially obscuring true biological differences. This is particularly relevant for the CSF pre-symptomatic comparison, which had the smallest and most imbalanced group in the dataset (Control, n = 4; Affected, n = 8). The limited number of significant proteins identified at this early stage may therefore reflect reduced power in the control group rather than a true absence of early proteomic change. The majority of CSF and tear proteins that were more abundant in the affected dogs, starting early in the disease progression, are characteristic components of neurons and likely represent the ongoing nervous system degeneration that starts early and continues throughout the course of the disease.

It is of particular interest that the protein that showed the greatest disease-related elevation in tears was ATP5BP, the beta subunit of the mitochondrial ATP synthase complex. A major component of the lysosomal storage material that accumulates in CLN2 disease and other forms of NCL is the subunit c protein of this same complex [43]. The marked increase in ATP5BP is therefore notable in the context of the established accumulation of mitochondrial ATP synthase components in NCL. ATP5BP was not the only mitochondrial protein altered in tears (Table 5). At the presymptomatic time point, additional components of the mitochondrial complex V (ATP5F1A and ATP5F1B) and complex I (NDUFA9) were increased, along with other mitochondrial proteins (i.e., HADHA, VDAC2, SLC25A5 and ACAT1). In contrast, no significantly altered components of complexes II, III, or IV were identified. Several intracellular and structural proteins were also altered in tear fluid (Supplemental Tables S10 and S11), indicating that cellular injury or turnover may contribute to the extracellular detection of mitochondrial proteins. However, ATP5BP exhibited a disproportionately large increase of nearly 1000-fold, substantially exceeding the changes observed for other mitochondrial proteins, and remained markedly elevated at the symptomatic stage. Thus, while cellular turnover or injury cannot be excluded as a contributing mechanism, cell lysis alone is unlikely to fully explain the magnitude of ATP5BP elevation. Together with the mitochondrial proteins identified in CSF, these findings support mitochondrial-associated proteins, particularly ATP5BP, as potential candidate biomarkers of CLN2 disease status.

Elevated CSF neurofilament light (NEFL) is a general biomarker for neurodegeneration, and it has already been shown that CSF levels of NEFL are a treatment-responsive biomarker in CLN2 disease [44,45]. Our data demonstrate that NEFL levels are elevated in CSF at symptomatic and advanced disease stages. Notably, increases in CSF NEFL were accompanied by correlated changes in multiple proteins in tears and plasma, indicating linked cross-biofluid associations as CLN2 disease progresses. These findings suggest that NEFL elevation is not an isolated event but occurs within a broader network of changes that extend beyond the central nervous system. While NEFL is a valuable marker of neuronal injury, a broader panel of complementary proteins reflecting additional aspects of disease biology (e.g., lysosomal dysfunction, mitochondrial stress, lipid metabolism, immune activation, and neuronal injury) may provide greater sensitivity and specificity for monitoring disease progression and therapeutic response. This concept is supported by a recent CSF biomarker discovery in CLN3 disease, which similarly identified combinations of neuronal, lysosomal, and inflammatory proteins that collectively discriminate disease status [46].

The only FDA-approved treatment for CLN2 disease is CNS enzyme replacement therapy [47]. This treatment involves regular infusions of recombinant TPP1 protein into the CSF via an implanted port. For these patients, regular CSF sampling for biomarker assessment is feasible because samples can be withdrawn via the same port used for infusions. Current trials are underway to assess the potential for one-time CSF gene therapy for CLN2 disease [3]. In this approach to treatment, there is no need for installation of permanent ports in affected children, which complicates the feasibility of regular CSF sampling. Thus, while CSF remains the most direct reflection of CNS pathology, fluid collection is invasive and may limit clinical applicability. One impactful aspect of this study is the evaluation of alternative biofluids. Collection of tear fluid is minimally invasive and establishes relevance in neurodegenerative disease research. The observed overlap between CSF and tear proteomic changes, particularly in lysosomal and immune-related proteins, supports the concept of shared molecular signatures across biofluids. This finding is consistent with prior studies demonstrating crosstalk between CSF and tear proteomes in neurological disease and suggests that tear-based biomarkers may be particularly valuable in longitudinal monitoring in populations where repeated sampling is required, and patients are amenable to collections [20,21,22,23].

Analysis of the blood plasma proteomes did not identify any strong candidate protein disease biomarkers. A possible reason for this is that blood plasma protein composition is regulated primarily by the liver, capillary endothelium, and the kidneys which together could quickly remove any aberrant proteins from the circulatory system. Thus, in this study, tear fluid and CSF appeared to be more useful biofluids for candidate biomarker identification compared to plasma.

Several interconnected biological themes emerged across biofluids and disease stages in CLN2-affected dogs. Altered abundance of lysosomal-associated proteins, including galectins, lysosomal hydrolases, and proteins involved in proteostasis, supports the presence of sustained lysosomal stress responses extending beyond the primary loss of TPP1 activity. This pattern is consistent with previous studies demonstrating that NCLs exhibit broad secondary changes in lysosomal proteins, reflecting compensatory remodeling of the lysosomal network rather than alterations limited to the primary defective enzyme. Proteomic analyses of brain, cerebrospinal fluid, and urine from CLN2 and other NCL models have similarly identified increased abundance of multiple lysosomal proteins together with activation of immune and inflammatory pathways, suggesting that these responses represent conserved features of disease progression [3,18,19,46,48,49]. The increased abundance of galectins observed in the present study is also particularly notable, as galectins function as intracellular sensors of lysosomal damage, initiating lysophagy and coordinating lysosomal repair, autophagic clearance, and lysosomal biogenesis [50]. These findings suggest that the lysosomal alterations detected across biofluids reflect ongoing activation of compensatory stress pathways in addition to impaired lysosomal degradation.

Similarly, proteins associated with mitochondrial function and energy metabolism were consistently altered, suggesting progressive disturbances in cellular bioenergetics and metabolic regulation, aligning with previous evidence that mitochondrial impairment is a key contributor to NCL pathogenesis [51,52,53]. More recent studies have further demonstrated that mitochondrial remodeling is closely linked to altered lipid metabolism and oxidative stress responses in NCL [54]. Changes in proteins related to lipid transport and membrane-associated processes further support disruption of pathways closely linked to lysosomal function and neuronal membrane homeostasis.

## 5. Conclusions

In summary, this study demonstrates that CLN2 disease is associated with coordinated, time-dependent proteomic alterations across multiple biofluids, reflecting interconnected disruptions in lysosomal function, mitochondrial metabolism, lipid homeostasis, and inflammatory response [49]. As an early-stage candidate biomarker discovery effort, these results should be interpreted with appropriate caution. The relatively small sample size necessitates validation in larger, independent cohorts, and orthogonal approaches such as targeted proteomics or immunoassays will be required to confirm the observed protein changes. Future studies will be essential to validate these candidates and determine their utility for tracking disease progression and assessing therapeutic response in human subjects.

## Figures and Tables

**Table 1:** Summary of samples analyzed. “X” denotes respective samples that were utilized. Samples not available are indicated by the cross mark/background.

			CSF (Ages in Months)			Plasma (Ages in Months)			Tears (Ages in Months)	
Dog ID	Genotype	Sex	Presymptomatic	Symptomatic	Late-Stage	Presymptomatic	Symptomatic	Late-Stage	Presymptomatic	Symptomatic
1	Carrier	F				X	X		X	X
2	Carrier	F	X	X		X	X	X	X	X
3	Carrier	F		X	X	X	X	X	X	X
4	Carrier	F					X	X	X	X
5	Carrier	F	X	X	X	X	X	X	X	X
6	Carrier	F	X	X	X	X	X	X	X	X
7	Carrier	M		X	X		X	X		
8	Carrier	M	X	X	X	X	X	X	X	X
9	Carrier	M			X		X	X		
10	Affected	F	X	X	X	X	X	X	X	X
11	Affected	F	X	X	X	X	X	X	X	X
12	Affected	M	X	X	X		X	X	X	X
13	Affected	M	X	X	X	X	X	X	X	X
14	Affected	M	X	X	X	X	X	X		
15	Affected	M	X	X	X	X	X	X	X	X
16	Affected	M	X	X	X	X	X	X		
17	Affected	M	X	X	X	X	X	X	X	X

**Table 2:** Differentially abundant proteins identified in CSF at both the symptomatic and late-stage time points in CLN2-affected dogs. Proteins meeting the significance criteria of raw *p*-value ≤ 0.05 and log2 fold change >0.585 or <−0.585 at both the symptomatic and late-stage time points are shown. Positive log2FC values indicate increased abundance in CLN2-affected dogs relative to age-matched controls, whereas negative log2FC values indicate decreased abundance.

Gene Name	Protein Name	Symptomatic (*p*-Value)	Late-Stage (*p*-Value)	Symptomatic (log2FC)	Late-Stage (log2FC)
TUBA4A	Tubulin alpha 4a	8.28 × 10^−6^	1.46 × 10^−3^	13.58	8.36
EPB41L1	Erythrocyte membrane protein band 4.1 like 1	1.40 × 10^−10^	2.61 × 10^−10^	9.72	9.53
SGIP1	SH3-containing GRB2-like protein 3-interacting protein 1	1.97 × 10^−5^	3.73 × 10^−5^	7.24	6.95
TOM1L2	Target of myb1 like 2 membrane trafficking protein	1.03 × 10^−3^	1.03 × 10^−3^	6.83	6.81
MAP6	Microtubule associated protein 6	1.85 × 10^−7^	7.84 × 10^−3^	5.34	2.28
CRKL	CRK like proto-oncogene	3.79 × 10^−3^	7.30 × 10^−3^	4.62	4.23
ADD1	Adducin 1	2.76 × 10^−2^	9.23 × 10^−5^	3.83	9.15
CNRIP1	CB1 cannabinoid receptor-interacting protein 1	1.84 × 10^−2^	6.67 × 10^−5^	3.62	8.18
ATP5IF1	ATPase inhibitor, mitochondrial	1.05 × 10^−4^	7.10 × 10^−4^	3.31	2.81
STIP1	Stress-induced-phosphoprotein 1	7.88 × 10^−4^	1.02 × 10^−2^	3.07	2.26
NEDD8	Ubiquitin-like protein NEDD8	4.97 × 10^−4^	1.18 × 10^−2^	3.00	2.08
LGALSL	Galectin	3.18 × 10^−2^	4.52 × 10^−2^	2.90	2.68
OTUB1	Ubiquitin thioesterase	5.50 × 10^−5^	1.13 × 10^−3^	2.90	2.28
CHI3L1	Chitinase-3-like protein 1	1.49 × 10^−25^	4.02 × 10^−37^	2.86	3.62
TREM2	Triggering receptor expressed on myeloid cells 2	6.57 × 10^−4^	1.77 × 10^−2^	2.83	1.88
PALM	Paralemmin 1	2.86 × 10^−2^	3.11 × 10^−2^	2.81	2.76
UCHL1	Ubiquitin carboxyl-terminal hydrolase	1.33 × 10^−4^	1.68 × 10^−3^	2.80	2.24
DYNC1I1	Dynein cytoplasmic 1 intermediate chain 1	2.49 × 10^−2^	3.81 × 10^−2^	2.58	2.36
MAPT	Microtubule associated protein tau	6.76 × 10^−6^	2.10 × 10^−3^	2.39	1.58
SNCB	Beta-synuclein	2.21 × 10^−13^	4.66 × 10^−7^	2.38	1.58
ST14	Matriptase	1.24 × 10^−2^	7.69 × 10^−3^	2.28	2.44
NEFL	Neurofilament light polypeptide	2.31 × 10^−2^	3.56 × 10^−2^	2.24	2.05
SYN1	Synapsin I	2.14 × 10^−3^	4.79 × 10^−2^	2.23	1.39
YWHAG	14-3-3 protein gamma	6.10 × 10^−7^	2.53 × 10^−9^	2.22	2.72
YWHAZ	14-3-3 protein zeta/delta	4.25 × 10^−9^	5.21 × 10^−12^	1.97	2.36
YWHAH	14-3-3 protein theta	1.11 × 10^−3^	9.72 × 10^−5^	1.97	2.39
ATOX1	Copper transport protein ATOX1	2.70 × 10^−3^	7.24 × 10^−3^	1.95	1.73
CALM2	Calmodulin 2	1.83 × 10^−4^	1.84 × 10^−3^	1.83	1.50
PFN2	Profilin	2.98 × 10^−2^	3.07 × 10^−2^	1.81	1.80
GAP43	Neuromodulin	7.59 × 10^−9^	1.43 × 10^−3^	1.80	0.96
PGAM1	Phosphoglycerate mutase	1.10 × 10^−6^	2.87 × 10^−7^	1.71	1.81
TXNDC17	Thioredoxin domain containing 17	1.46 × 10^−2^	4.07 × 10^−2^	1.69	1.40
HSP90AA1	Heat shock protein HSP 90-alpha	2.54 × 10^−2^	4.37 × 10^−2^	1.66	1.49
PABPC1L	Poly(A) binding protein cytoplasmic 1 like	3.28 × 10^−6^	1.76 × 10^−8^	1.66	2.05
MAP2	Microtubule-associated protein 2	5.63 × 10^−3^	3.12 × 10^−2^	1.53	1.18
MAP1B	Microtubule associated protein 1B	2.65 × 10^−2^	9.39 × 10^−6^	1.49	3.23
DYNLL2	Dynein light chain	1.05 × 10^−10^	5.61 × 10^−7^	1.49	1.14
WDR1	WD repeat domain 1	7.16 × 10^−7^	5.03 × 10^−5^	1.38	1.12
LOC102152706	Thymosin beta	8.93 × 10^−10^	1.56 × 10^−9^	1.38	1.36
YWHAE	14-3-3 protein epsilon	2.23 × 10^−2^	5.52 × 10^−4^	1.32	2.05
TXN	Thioredoxin	2.07 × 10^−3^	3.21 × 10^−2^	1.30	0.90
PEBP1	Phosphatidylethanolamine-binding protein 1	4.99 × 10^−13^	5.47 × 10^−14^	1.26	1.32
SH3BGRL3	SH3 domain-binding glutamic acid-rich-like protein	5.92 × 10^−7^	4.04 × 10^−7^	1.15	1.17
ENO2	Enolase 2	1.16 × 10^−11^	1.79 × 10^−7^	1.13	0.86
CKB	Creatine kinase B-type	4.25 × 10^−4^	4.44 × 10^−12^	1.12	2.32
LGALS1	Galectin 1	1.28 × 10^−5^	3.44 × 10^−4^	1.12	0.91
STMN1	Stathmin	1.04 × 10^−2^	2.08 × 10^−6^	1.09	2.11
PPIC	Peptidyl-prolyl cis-trans isomerase	3.00 × 10^−2^	3.47 × 10^−2^	1.07	1.05
GDI1	Rab GDP dissociation inhibitor	1.34 × 10^−4^	1.06 × 10^−3^	1.05	0.90
RTN1	Reticulon	4.34 × 10^−2^	9.39 × 10^−3^	1.01	1.30
PCP4	Purkinje cell protein 4	1.33 × 10^−3^	7.22 × 10^−3^	0.96	0.80
TKT	Transketolase	6.59 × 10^−6^	2.75 × 10^−4^	0.88	0.71
CFL1	Cofilin 1	1.58 × 10^−4^	2.55 × 10^−4^	0.87	0.85
ALDOC	Fructose-bisphosphate aldolase	2.42 × 10^−6^	5.39 × 10^−23^	0.87	1.87
PGAM2	Bisphosphoglycerate mutase	3.19 × 10^−2^	2.13 × 10^−2^	0.81	0.88
C1QA	Complement C1q A chain	1.71 × 10^−2^	1.55 × 10^−4^	0.77	1.24
DBI	Acyl-CoA-binding protein	1.31 × 10^−4^	5.33 × 10^−7^	0.76	1.01
ITIH4	Inter-alpha-trypsin inhibitor heavy chain 4	1.08 × 10^−2^	1.28 × 10^−2^	0.76	0.74
IGSF8	Immunoglobulin superfamily member 8	1.95 × 10^−5^	5.43 × 10^−4^	0.74	0.60
CYCS	Cytochrome c	1.46 × 10^−3^	3.69 × 10^−7^	0.69	1.11
RARRES2	Retinoic acid receptor responder protein 2	1.04 × 10^−2^	5.68 × 10^−3^	0.67	0.73
VSIG4	V-set and immunoglobulin domain containing 4	7.97 × 10^−3^	1.42 × 10^−4^	0.62	0.89
CCL23	CCL23	3.68 × 10^−2^	1.77 × 10^−3^	0.59	0.88
NPTX1	Neuronal pentraxin 1	1.27 × 10^−3^	2.09 × 10^−12^	-0.59	−1.30
PCSK1N	Proprotein convertase subtilisin/kexin type 1 inhibitor	5.99 × 10^−4^	3.78 × 10^−6^	−0.60	−0.81
INAFM1	InaF motif containing 1	1.52 × 10^−2^	2.50 × 10^−4^	−0.61	−0.93
OXT	Oxytocin-neurophysin 1	1.28 × 10^−3^	4.91 × 10^−4^	−0.65	−0.70
FGFR1	Fibroblast growth factor receptor 1	4.29 × 10^−3^	5.09 × 10^−3^	−0.67	−0.66
VGF	VGF nerve growth factor inducible	2.92 × 10^−6^	4.65 × 10^−9^	−0.70	−0.88
PTPRN	Protein tyrosine phosphatase receptor type N	1.65 × 10^−5^	3.35 × 10^−8^	−0.78	−1.00
FAM3C	Protein FAM3C	2.10 × 10^−5^	2.41 × 10^−7^	−0.79	−0.96
CSPG5	Chondroitin sulfate proteoglycan 5	2.18 × 10^−2^	1.40 × 10^−2^	−0.86	−0.93
RELN	Reelin	2.82 × 10^−2^	9.23 × 10^−6^	−0.88	−1.80
MANSC1	MANSC domain containing 1	9.19 × 10^−3^	4.03 × 10^−2^	−0.89	−0.69
TNFRSF21	TNF receptor superfamily member 21	1.15 × 10^−2^	4.72 × 10^−3^	−0.92	−1.03
CCK	Cholecystokinin	3.15 × 10^−2^	9.51 × 10^−4^	−0.97	−1.50
ARSA	Arylsulfatase A	5.40 × 10^−3^	1.12 × 10^−2^	−1.03	−0.94
ISM2	Isthmin 2	4.31 × 10^−2^	8.88 × 10^−3^	−1.13	−1.47
PDYN	Proenkephalin-B	4.34 × 10^−3^	1.06 × 10^−2^	−1.18	−1.06
PRRT3	Proline rich transmembrane protein 3	8.93 × 10^−6^	3.32 × 10^−3^	−1.28	−0.84
AJAP1	Adherens junctions associated protein 1	4.09 × 10^−3^	3.27 × 10^−4^	−1.33	−1.68
PCSK1	Neuroendocrine convertase 1	1.48 × 10^−3^	1.54 × 10^−3^	−1.33	−1.32
SST	Somatostatin	1.25 × 10^−3^	2.60 × 10^−2^	−1.38	−0.94
TMEM59L	Transmembrane protein 59 like	1.97 × 10^−4^	5.30 × 10^−3^	−2.16	−1.60
ALB	Albumin	1.12 × 10^−4^	3.82 × 10^−2^	−2.36	−1.24

**Table 3:** Differentially abundant proteins in plasma from CLN2-affected dogs. List of proteins with raw *p* ≤ 0.05 and log2 fold change >0.585 or <−0.585 at each time point. Proteins are grouped by direction of change relative to age-matched controls.

	log2FC	*p*-Value	FDR	Protein Name	Gene Name
**Presymptomatic**	**Up-regulated proteins**				
	1.24	2.40 × 10^−3^	1.14 × 10^−1^	Insulin-like growth factor-binding protein 5	IGFBP5
	1.21	2.24 × 10^−4^	1.85 × 10^−2^	Adiponectin, C1Q and collagen domain containing	ADIPOQ
	1.19	1.89 × 10^−2^	3.05 × 10^−1^	Maltase-glucoamylase 2 (putative)	MGAM2
	1.01	1.24 × 10^−3^	8.24 × 10^−2^	Cytochrome c	CYCS
	0.74	2.71 × 10^−2^	3.57 × 10^−1^	Inter-alpha-trypsin inhibitor heavy chain 4	ITIH4
	0.63	1.51 × 10^−2^	2.94 × 10^−1^	Cadherin-1	CDH1
	0.63	1.13 × 10^−2^	2.64 × 10^−1^	Acid sphingomyelinase-like phosphodiesterase	SMPDL3A
	**Down-regulated proteins**				
	−2.52	1.64 × 10^−5^	5.43 × 10^−3^	Ig-like domain-containing protein	Unknown
	−2.24	3.62 × 10^−5^	6.00 × 10^−3^	Immunoglobulin V-set domain-containing protein	VSIG
	−1.01	3.20 × 10^−3^	1.19 × 10^−1^	Ig-like domain-containing protein	Unknown
	−0.99	2.86 × 10^−2^	3.57 × 10^−1^	Ig-like domain-containing protein	Unknown
	−0.90	6.38 × 10^−3^	1.92 × 10^−1^	Uncharacterized protein	Unknown
	−0.83	4.67 × 10^−2^	4.68 × 10^−1^	Transforming growth factor beta induced	TGFBI
	−0.82	1.93 × 10^−2^	3.05 × 10^−1^	Ig-like domain-containing protein	Unknown
	−0.80	4.29 × 10^−2^	4.58 × 10^−1^	Vimentin	VIM
	−0.77	2.23 × 10^−4^	1.85 × 10^−2^	Complement factor properdin	CFP
	−0.71	4.03 × 10^−2^	4.45 × 10^−1^	Hemoglobin subunit alpha1	HBQ1
	−0.70	9.91 × 10^−3^	2.52 × 10^−1^	Ig heavy chain V region MOO	IGHV
	−0.67	1.52 × 10^−2^	2.94 × 10^−1^	Fetuin B	FETUB
	−0.66	4.62 × 10^−2^	4.68 × 10^−1^	Serine peptidase inhibitor	SPINT1
	−0.65	2.48 × 10^−2^	3.57 × 10^−1^	Ig-like domain-containing protein	Unknown
**Symptomatic**	**Up-regulated proteins**				
	8.12	4.55 × 10^−2^	4.40 × 10^−1^	Beta-casein	CSN2
	3.12	1.62 × 10^−2^	2.68 × 10^−1^	Serum amyloid A protein	SAA1
	1.45	8.57 × 10^−4^	4.73 × 10^−2^	Maltase-glucoamylase 2	MGAM2
	1.38	7.21 × 10^−5^	2.32 × 10^−2^	Insulin-like growth factor-binding protein 5	IGFBP5
	1.21	5.42 × 10^−3^	1.49 × 10^−1^	Secreted phosphoprotein 24	SPP2
	1.00	2.18 × 10^−3^	9.03 × 10^−2^	Proteasome subunit alpha type	PSMA7
	0.94	2.71 × 10^−2^	3.58 × 10^−1^	Kininogen 1	KNG1
	0.89	8.41 × 10^−4^	4.73 × 10^−2^	Cytochrome c	CYCS
	0.86	5.96 × 10^−4^	4.73 × 10^−2^	Insulin like growth factor binding protein 7	IGFBP7
	0.84	3.60 × 10^−3^	1.32 × 10^−1^	Lipopolysaccharide-binding protein	LBP
	0.70	1.47 × 10^−2^	2.57 × 10^−1^	Inter-alpha-trypsin inhibitor heavy chain 4	ITIH4
	0.63	1.23 × 10^−2^	2.56 × 10^−1^	CD44 antigen	CD44
	**Down-regulated proteins**				
	−1.22	1.39 × 10^−2^	2.56 × 10^−1^	Ig-like domain-containing protein	LOC612180
	−1.17	1.38 × 10^−3^	6.53 × 10^−2^	Thymosin beta	LOC102152706
	−1.08	2.36 × 10^−4^	2.61 × 10^−2^	Kunitz-type protease inhibitor 1	SPINT1
	−0.88	1.35 × 10^−2^	2.56 × 10^−1^	Leukocyte cell-derived chemotaxin 1	CNMD
	−0.82	4.61 × 10^−2^	4.40 × 10^−1^	Microfibril associated protein 5	MFAP5
	−0.81	1.91 × 10^−2^	2.88 × 10^−1^	Fibroleukin	FGL2
	−0.75	4.60 × 10^−2^	4.40 × 10^−1^	Collagen alpha-1(I) chain	COL1A1
	−0.74	1.40 × 10^−4^	2.32 × 10^−2^	Insulin-like growth factor-binding protein 2	IGFBP2
	−0.63	1.02 × 10^−2^	2.56 × 10^−1^	Fetuin B	FETUB
**Late-Stage**	**Up-regulated proteins**				
	1.65	4.87 × 10^−2^	3.84 × 10^−1^	Haptoglobin	HP
	1.64	1.34 × 10^−2^	2.78 × 10^−1^	Cytokine like 1	CYTL1
	1.41	6.62 × 10^−3^	1.79 × 10^−1^	Rerating islet-derived 3 alpha	REG3A
	1.14	8.78 × 10^−4^	6.13 × 10^−2^	Apolipoprotein E	APOE
	1.03	6.69 × 10^−4^	6.13 × 10^−2^	Lipopolysaccharide-binding protein	LBP
	0.84	5.00 × 10^−3^	1.66 × 10^−1^	Inter-alpha-trypsin inhibitor heavy chain 4	ITIH4
	0.82	1.53 × 10^−3^	8.44 × 10^−2^	Insulin like growth factor binding protein 7	IGFBP7
	0.81	3.66 × 10^−2^	3.55 × 10^−1^	Ig-like domain-containing protein	Unknown
	0.74	3.36 × 10^−2^	3.48 × 10^−1^	Ig-like domain-containing protein	Unknown
	0.64	3.57 × 10^−3^	1.31 × 10^−1^	Ig-like domain-containing protein	Unknown
	0.62	2.62 × 10^−2^	3.24 × 10^−1^	Cadherin-5	CDH5
	**Down-regulated proteins**				
	−9.64	2.70 × 10^−2^	3.24 × 10^−1^	Chloride channel accessory 4	CLCA4
	−7.62	4.77 × 10^−2^	3.84 × 10^−1^	Keratin 36	KRT36
	−2.23	3.24 × 10^−2^	3.48 × 10^−1^	Cadherin 17	CDH17
	−1.10	7.03 × 10^−3^	1.79 × 10^−1^	Ig-like domain-containing protein	Unknown
	−0.87	9.27 × 10^−4^	6.13 × 10^−2^	Carboxylic ester hydrolase	GTF3C5
	−0.85	2.72 × 10^−2^	3.24 × 10^−1^	Collagen alpha-1(I) chain	COL1A1
	−0.76	1.33 × 10^−4^	2.21 × 10^−2^	Insulin-like growth factor-binding protein 2	IGFBP2
	−0.65	5.68 × 10^−3^	1.71 × 10^−1^	Cartilage oligomeric matrix protein	COMP
	−0.65	3.12 × 10^−3^	1.29 × 10^−1^	Platelet-derived growth factor receptor beta	PDGFRB
	−0.64	2.93 × 10^−2^	3.34 × 10^−1^	Kunitz-type protease inhibitor 1	SPINT1
	−0.59	1.31 × 10^−2^	2.78 × 10^−1^	ADP-ribosyl cyclase/cyclic ADP-ribose hydrolase	BST1

**Table 4:** Major biological themes identified through cross-biofluid correlation analysis. Representative proteins participating in significant pairwise correlations between CSF, tear, and plasma samples grouped according to predominant biological functions.

Representative Proteins	Biological Relevance
ADD1, ADD2, MAP1B, MAP2, MAP6, MAPT, TUBA4A, TUBB3, CFL1, EPB41L1, PFN2, TPM1, WDR1	Neuronal structure, cytoskeletal stability, axonal integrity
SYN1, STXBP1, PACSIN1, SGIP1, TOM1L2, GAP43, NCAM1, CNRIP1, PCP4, PDYN, SST, OXT	Synaptic transmission, vesicle recycling, neuronal communication
DYNC1I1, DYNC1LI2, DYNLL2	Microtubule-based intracellular transport and axonal trafficking
CTSF, GALNS, GNS, LGALS1, LGALS9, NPC2, PSMB7, OTUB1, STIP1, UCHL1, HSP90AA1	Lysosomal function, protein turnover, cellular stress responses
APOE, FETUB, LBP, ADIPOQ, PON2, TTR	Lipid homeostasis, transport, and metabolic regulation
C1QA, VSIG4, TREM2, TNFRSF21, RARRES2, CCL23, CHI3L1	Innate immunity, neuroinflammation, immune signaling

**Table 5:** Mitochondrial proteins significantly altered in tear fluid from CLN2-affected dogs. Proteins associated with mitochondrial respiratory chain complexes I–V and other mitochondrial proteins identified at the presymptomatic and symptomatic disease stages are shown. Values indicate log2 fold changes in affected dogs relative to age-matched unaffected controls. Arrows indicate direction of change. “None identified” indicates that no proteins associated with the indicated mitochondrial complex met the predefined significance and fold change criteria (raw *p* < 0.05 and log2 fold change >0.585 or <−0.585).

Mitochondrial Complex	Presymptomatic	Symptomatic
I	NDUFA9 ↑ 3.75	None identified
II	None identified	None identified
III	None identified	None identified
IV	None identified	None identified
V	ATP5PB ↑ 9.99, ATP5F1A ↑ 0.95, ATP5F1B ↑ 0.79	ATP5PB **↑** 9.65
Other mitochondrial proteins	SLC25A5 ↑ 1.80, VDAC2 ↑ 1.34, ACAT1 ↑ 0.77, HADHA ↑ 0.72	YRDC ↑ 0.64

## Data Availability

The original contributions presented in this study are included in the article/Supplementary Materials. Further inquiries can be directed to the corresponding author.

## Supplementary Materials

The following supporting information can be downloaded at: https://www.mdpi.com/article/10.3390/biology15191761/s1, Supplementary Figure S1: Circos Plot, Supplementary Figure S2: Correlation Heatmap, and Supplemental File. The mass spectrometry proteomics data have been deposited to the ProteomeXchange Consortium via the PRIDE partner repository with the dataset identifier [place holder]. Table S1: Differentially Abundant Proteins in CSF_presymptomatic. Table S2: Differentially Abundant Proteins in CSF_symptomatic. Table S3: Differentially Abundant Proteins in CSF_late-stage. Table S4: Functional Enrichment Analysis Results_CSF_presymptomatics. Table S5: Functional Enrichment Analysis Results_CSF_symptomatics. Table S6: Functional Enrichment Analysis Results_CSF_late-stages. Table S7: Functional Enrichment Analysis Results_Plasma_presymptomatic. Table S8: Functional Enrichment Analysis Results_Plasma_symptomatic. Table S9: Functional Enrichment Analysis Results_Plasma_late-stage. Table S10: Differentially Abundant Proteins in Tears_presymptomatic. Table S11: Differentially Abundant Proteins in Tears_symptomatic. Table S12: Functional Enrichment Analysis Results_Tear_presymptomatic. Table S13: Functional Enrichment Analysis Results_Tear_symptomatic. Table S14: Circos Plot_Pairwise Correlations.

## Abbreviations

The following abbreviations are used in this manuscript:

NCL	Neuronal ceroid lipofuscinosis
CLN2	Ceroid lipofuscinosis, neuronal 2
TPP1	Tripeptidyl peptidase 1
CSF	Cerebrospinal fluid
CNS	Central nervous system
