# Supplementary material for: Biofluid Proteomic Biomarker Discovery in a Large Animal Model of CLN2 Neuronal Ceroid Lipofuscinosis
Source: Biology (Basel). 2026 Oct 2;15(19):1761. doi: 10.3390/biology15191761 (PMC13663543; doi:10.3390/biology15191761)
Supplement: Supplementary file 1 [file biology-15-01761-s001.zip › Supplemental Figure S1_Circos Plot.pdf]

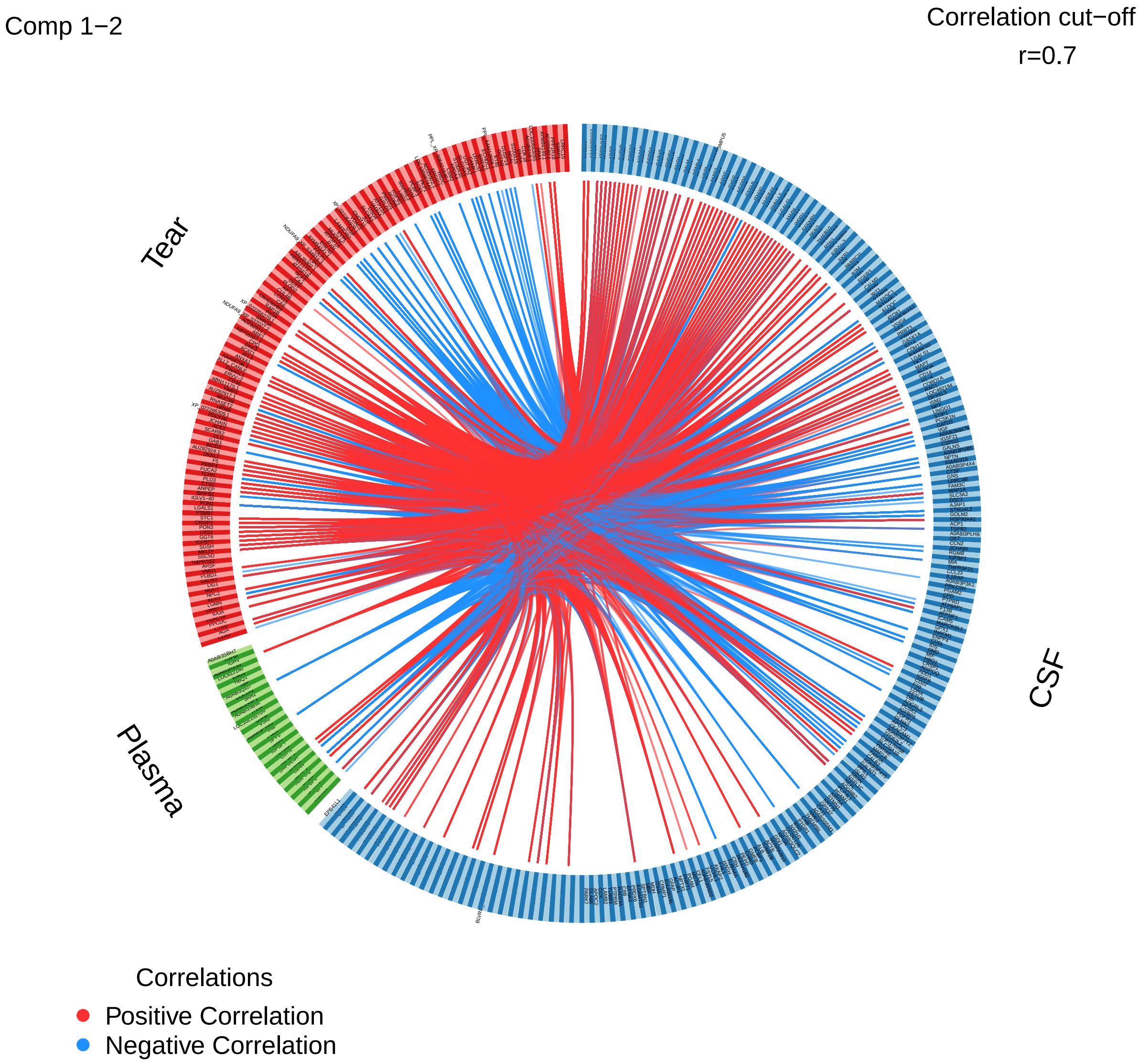
Comp 1−2
Correlation cut−off
r=0.7
Tear
CSF
Plasma
Correlations
Positive Correlation
Negative Correlation
